# Calorie Restriction Rescues Mitochondrial Dysfunction in Adck2-Deficient Skeletal Muscle

**DOI:** 10.3389/fphys.2022.898792

**Published:** 2022-07-14

**Authors:** Juan Diego Hernández-Camacho, Daniel J. M. Fernández-Ayala, Cristina Vicente-García, Ignacio Navas-Enamorado, Guillermo López-Lluch, Clara Oliva, Rafael Artuch, Judith Garcia-Villoria, Antonia Ribes, Rafael de Cabo, Jaime J. Carvajal, Plácido Navas

**Affiliations:** ^1^ Centro Andaluz de Biología del Desarrollo, Universidad Pablo de Olavide-CSIC-JA, Sevilla, Spain; ^2^ CIBERER, Instituto de Salud Carlos III, Madrid, Spain; ^3^ Atsena Therapeutics, Durham, NC, United States; ^4^ Clinical Biochemistry Department, Institut de Recerca Sant Joan de Déu, Barcelona, Spain; ^5^ Inborn Errors of Metabolism Section, Biochemistry and Molecular Genetics Department, Hospital Clinic, Barcelona, Spain; ^6^ Translational Gerontology Branch, National Institute on Aging Intramural Research Program, National Institutes of Health, Baltimore, MD, United States

**Keywords:** mitochondria, coenzyme Q, food deprivation, metabolism, fatty acids

## Abstract

ADCK2 haploinsufficiency-mediated mitochondrial coenzyme Q deficiency in skeletal muscle causes mitochondrial myopathy associated with defects in beta-oxidation of fatty acids, aged-matched metabolic reprogramming, and defective physical performance. Calorie restriction has proven to increase lifespan and delay the onset of chronic diseases associated to aging. To study the possible treatment by food deprivation, heterozygous *Adck2* knockout mice were fed under 40% calorie restriction (CR) and the phenotype was followed for 7 months. The overall glucose and fatty acids metabolism in muscle was restored in mutant mice to WT levels after CR. CR modulated the skeletal muscle metabolic profile of mutant mice, partially rescuing the profile of WT animals. The analysis of mitochondria isolated from skeletal muscle demonstrated that CR increased both CoQ levels and oxygen consumption rate (OCR) based on both glucose and fatty acids substrates, along with mitochondrial mass. The elevated aerobic metabolism fits with an increase of type IIa fibers, and a reduction of type IIx in mutant muscles, reaching WT levels. To further explore the effect of CR over muscle stem cells, satellite cells were isolated and induced to differentiate in culture media containing serum from animals in either *ad libitum* or CR diets for 72 h. Mutant cells showed slower differentiation alongside with decreased oxygen consumption. *In vitro* differentiation of mutant cells was increased under CR serum reaching levels of WT isolated cells, recovering respiration measured by OCR and partially beta-oxidation of fatty acids. The overall increase of skeletal muscle bioenergetics following CR intervention is paralleled with a physical activity improvement, with some increases in two and four limbs strength tests, and weights strength test. Running wheel activity was also partially improved in mutant mice under CR. These results demonstrate that CR intervention, which has been shown to improve age-associated physical and metabolic decline in WT mice, also recovers the defective aerobic metabolism and differentiation of skeletal muscle in mice caused by ADCK2 haploinsufficiency.

## Introduction

Calorie restriction (CR) is the most robust dietary intervention that promotes healthspan and longevity, decreasing the incidence of age-related diseases (De Cabo et al., 2014; Balasubramanian et al., 2017). Reduced calorie intake is not the only driver of health and ageing benefits, as fasting periods between meals have been shown to be essential for enhancing stress resistance, improving glucose metabolism and promoting recycling of damaged molecules (Di Francesco et al., 2018; Fatahi et al., 2020; Hofer et al., 2022). CR induces a metabolic adaptation leading to coordinate bioenergetics between lipid and glucose catabolism and, to some extent, to the catabolism of branched-chain amino acids (López-Lluch and Navas, 2016; Collet et al., 2017; Most and Redman, 2020). CR induces benefits in ageing mammals, dependent on genetic background, gender and the total energy intake (Mitchell et al., 2016; Ingram and De Cabo, 2017). Basically, these benefits consist on the metabolic adaptation of tissues and organs in order to maintain energy homeostasis by bioenergetic substrate selection in feeding and fasting periods, a process known as metabolic flexibility (Smith et al., 2018). During CR, the nutrient-sensing pathways are adapted to reduce glucose oxidation and increase fatty acids oxidation. This switch is activated by the energy sensor adenosine monophosphate-activated protein kinase (AMPK), in response to the increase in the AMP/ATP ratio, while carnitine palmitoyl transferase-1 (CPT-1) is also activated by AMPK inhibition of acetyl-CoA carboxylase (ACC). Additionally, the rise in fatty acids oxidation induces the citrate-dependent inhibition of phosphofructokinase and GLUT4, reducing glucose catabolism (Muoio, 2014; Spriet, 2014; Smith et al., 2018; González et al., 2020). Increased AMP/ATP or NAD^+^/NADH ratios also contribute to activate PGC1α and FOXOs by phosphorylation and deacetylation. Both ratios inhibit the mTOR pathway, which increases both autophagy and mitochondrial biogenesis, and blocks protein synthesis (Madeo et al., 2015; Smith et al., 2018; Sorrentino et al., 2018; Yu et al., 2019). All these pathways ultimately target mitochondria, which can select bioenergetics fuel under dietary interventions (Kuzmiak-Glancy and Willis, 2014; Muoio, 2014; Wang et al., 2022).

Mitochondrial function decline is a hallmark of ageing (López-Otín et al., 2013; López-Otín et al., 2016), impairing cellular activities, and compromising the cellular ability of metabolic flexibility, leading to weakness, frailty and disability (Gonzalez-Freire et al., 2015), impacting upon mitochondrial content and bioenergetics efficiency (Tieland et al., 2018; Sharma et al., 2019). Mitochondrial dysfunction is also responsible for skeletal muscle strength loss, decreasing physical performance (Zane et al., 2017), and promoting skeletal muscle disease and sarcopenia (Faitg et al., 2017; Migliavacca et al., 2019). CR restores mitochondria content and efficiency in skeletal muscle by increasing mitochondrial biogenesis activating PGC1α through the AMPK/sirtuins pathway and modulating of mitochondria through SIRT3-dependent acetylome (López-Lluch et al., 2006; Hebert et al., 2013; Martin-Montalvo and De Cabo, 2013). Therefore, CR could prevent the decrease of muscle cross-sectional area, sarcopenic development and muscle loss associated with ageing in the elderly (Börsch et al., 2021; Mizunoe et al., 2021). Other factors also contribute to healthspan and longevity. For example, nicotinamide dependent NAD^+^ increase induces health span without affecting lifespan (Mitchell et al., 2018) and mTORC2 is required for insulin homeostasis but is dispensable for health- and lifespan (Yu et al., 2019).

Coenzyme Q (CoQ) deficiency syndrome includes a highly heterogeneous group of mitochondrial diseases that show low levels of CoQ in tissues and organs caused by either a defect in the CoQ biosynthesis pathway, or in another mitochondrial specific pathway. CoQ-deficiency patients show symptoms ranging from high severity early-onset disease, to moderate severity late-onset disease (Alcázar-Fabra et al., 2021; Laugwitz et al., 2021; Naess et al., 2021; Navas et al., 2021). The major consequence of this deficiency is mitochondrial dysfunction with low levels of ATP production (Navas et al., 2021). However, there are other CoQ-dependent redox reactions in the respiratory chain that condition cellular metabolism (Alcázar-Fabra et al., 2018; Banerjee et al., 2021) as well as extramitochondrial CoQ-linked cytosolic redox reactions that contribute to CoQ deficiency pathogenesis and cellular homeostasis (Gueguen et al., 2021; Hernández-Camacho et al., 2021). We hypothesize that mild conditions of CoQ deficiency could be rescued by CR through an improvement of mitochondrial biogenesis and efficiency, and physical activity and metabolism, reversing the pathological phenotype.

*ADCK* genes encode members of the aarF domain-containing mitochondrial protein kinases, which are a family of proteins related to CoQ biosynthesis and maintenance, and linked to mitochondrial disorders affecting specific tissues/organs. Several mutations in *ADCK* genes have been reported: *ADCK3, mutations* cause autosomal recessive cerebellar ataxia (Traschütz et al., 2020), while mutations in *ADCK4* promote CoQ-responsive, steroid-resistant nephrotic syndrome (Ashraf et al., 2013); *ADCK2* haploinsufficiency causes adult-onset myopathy with CoQ deficiency in skeletal muscle, associated with severe impairment of physical activity, and an overall defect in mitochondrial lipid metabolism. This phenotype is recapitulated in a heterozygous *Adck2* knockout mouse model (Vázquez-Fonseca et al., 2019), which only partially responds to CoQ supplementation. Here, we demonstrate that a long-term CR intervention in *Adck2* ^*+/−*^ mice recovers overall glucose and lipid homeostasis, enhances physical activity, increases mitochondrial function and aerobic metabolism in skeletal muscle, and improves functionality of satellite cells. We propose that CR should be considered as an alternative strategy for the treatment of nuclear-dependent mild phenotypes of CoQ deficiency syndrome.

## Materials and Methods

### Mouse Model, Study Approval, and Calorie Restriction Conditions

Mouse housing and tissue collection were performed using protocols approved by the Universidad Pablo de Olavide Ethics Committee (Seville, Spain; authorization no 12/03/2021/033) in accordance with Spanish Royal Decree 53/2013, European Directive 2010/63/EU, and other relevant guidelines. Mice were bred and raised in the animal facility at the Centro Andaluz de Biología del Desarrollo (CABD). The *Adck2* knockout mouse model was generated in the C57BL/6J background at the University of Michigan Transgenic Animal Model Core, Biomedical Research Core Facilities (Ann Arbor, MI, United States) as previously described (Vázquez-Fonseca et al., 2019). Mice were individually housed in autoclavable polycarbonate cages ensuring access to food under the calorie restriction regime. Environmental conditions of housing were 12 h light/dark cycle. Start time for the light period was 08:00 a.m. Temperature and humidity oscillated within the range of 22°C ± 2°C and 54 ± 1%, respectively. The diet of mice was based on SAFE D40 (Scientific Animal Food and Engineering) (composition: cereals 84.6%, vitamins and minerals 3.9%, and vegetable proteins 11.5%); mice received autoclaved water *ad libitum*. Diet information is detailed in Supplementary Figure S1A. Mice were sacrificed by cervical dislocation.

Mice on CR were subjected to a 40% reduction in daily food intake, establishing a regimen by which 60% of *ad libitum* diet was added daily to the cages at 09:00 a.m. Basal food intake was determined over 2 months before CR, and it was established to be approximately 4 g per day. Therefore, mice on CR were fed 2.4 g daily of a standard diet for laboratory mice. For the CR experiment, 6 month-old male mice were maintained on CR for 7 months to induce long-term adaptation to food deprivation. Adult mice (6 month-old) were selected for the experiment instead of younger mice to avoid negative effects upon growth and development. Offspring from heterozygous *Adck2* male and female mice were used for experiments. Age-matched male littermates fed *ad libitum* were used as controls.

### Glucose and Insulin Tests

Both fasting glucose and insulin levels were measured after the mice had fasted 12 h (08:00 p.m.–08:00 a.m.). Glucose was measured in blood samples with an Accu Chek Performa glucometer, (Basel, Switzerland). To measure insulin levels, blood samples were collected into heparin-treated tubes and centrifuged at 2,000 g for 10 min at 4°C to isolate the plasma fraction from the transparent upper phase. Insulin levels were analyzed using a Mouse Insulin Elisa kit (Mercodia 10-1247-01/10-1247-10, AD Bioinstruments, Barcelona, Spain). Insulin resistance was calculated from the Homeostatic model assessment (HOMA) index using the formula: fasting insulin (µU/L) x fasting glucose (nmol/L)/22.5 (HOMA2 Calculator, University of Oxford, https://www.dtu.ox.ac.uk/homacalculator/). For the glucose tolerance test (GTT), mice were first fasted for 12 h (08:00 p.m.–08:00 a.m.) and then received an intraperitoneal injection of glucose (2 g/kg weight) using a solution of 200 mg/ml D-glucose. Glucose measurements were taken at 15-min intervals for the first hour and every 30 min for the second hour. Insulin homeostasis after glucose load was also analyzed after this 12-h fasting period by quantifying insulin levels at 20 and 40 min after glucose injection.

For the insulin tolerance test (ITT), mice were fasted for 5 h (10:00 a.m.–03:00 p.m.) and then received an intraperitoneal injection of insulin (0.75 U/kg weight); glucose measurements were taken at 15-min intervals for 1 h. Gluconeogenesis was evaluated by the pyruvate tolerance test (PTT). Mice were fasted for 5 h (10:00 a.m.–03:00 p.m.) and then received an intraperitoneal injection of pyruvate (2 g/kg weight); glucose measurements were taken at 15-min intervals for the first hour and every 30 min for the second hour.

### Physical Performance Assessment

All tests were started at the same time of the day (2:00 p.m.) to reduce variability associated with circadian rhythms. For CR mice testing, food was added to the cage at 09:00 a.m. and test was started at 2.00 p.m. All mice were brought to the experimental room for acclimatization 30 min before starting any functional tests.

The grip strength test was used to evaluate *in vivo* muscle force (Grip Strength Columbus apparatus, Columbus Instruments, OH, United States) (Aartsma-Rus and van Putten, 2014). Mice were held by the middle of the tail and lowered over the grid, to allow them to grasp it tightly with the forelimbs or with all limbs. Every mouse was tested 5 times and the three most similar values used for data analysis.

In the weight-lifting assay (Deacon, 2013), the mice were held by the middle of the tail and allowed to grasp steel mesh balls attached to increasingly heavier weights, scoring a value depending on the weight (16 g, value = 1; 32 g, value = 2; 48 g, value = 3; 64 g, value = 4; and 80 g, value = 5). The test was considered as passed after 3 s and a new, heavier weight was offered to the mouse, whereas if mice dropped the weight before 3 s, the total time (in seconds) was noted. Mice were allowed 3 attempts to pass the test with each weight, giving to each weight the greatest number of seconds without dropping it. The final score was calculated as the product of the value for the heaviest weight held for more than 1 s, multiplied by the time (seconds), and normalized to body weight.

The treadmill exercise test was used to determine mice aerobic capacity. Mice were forced to run until exhaustion on a 10° uphill open treadmill using a mild electric shock (Treadmill Columbus 1055M-E50; Columbus Instruments, OH, United States (Dougherty et al., 2016; Richardson et al., 2016; Rocchi and He, 2017). Mice were acclimatized for 2 days, with a regime of 5 min running at 8 m/min, and an additional 5 min at 10 m/min the second day. For the test, mice underwent a single bout of running for 60 min, starting at 10 m/min for 20 min and increasing the speed by 1 m/min every 4 min for the remaining 40 min.

### Automated Phenotyping

An automated home cage phenotyping system (PhenoMaster TSE system, TSE Systems GmbH, Hessen, Germany) was used to further characterize the mouse phenotype under controlled conditions (Fernández-Calleja et al., 2018). The phenotyping system allows to record *in vivo* parameters during day and night periods. Several experiments were performed using always the same starting and duration times. Mice from all the groups were included in every experiment. The first 36 h from every experiment were considered acclimatization period and were discarded. Data from day/night cycles from the different mice were used for the analysis.

### Analysis of CoQ Levels, Acyl-Carnitines, β-Hydroxybutyrate, Free Fatty Acids and Metabolomics

Mitochondria from skeletal muscle were isolated through serial centrifugations under osmotic conditions as previously described (Hernández-Camacho et al., 2022). Pellets of 100 µg of protein of enriched mitochondria solution were resuspended in 170 µl of phosphate-buffered saline (PBS) and 10 µl of 10 µM CoQ_6_ were included as an internal loss control. Sodium dodecyl sulphate (SDS) was added to a final concentration of 1% (w/v) to rupture cellular membranes and the mixture vortexed for 1 min. Afterwards, 300 µ1 of ethanol-isopropanol (90:10v/v) were added and vortexed for 1 min. 700 µl of hexane were added, vortexed for 1 min, and followed by centrifugation at 1000 g for 5 min at 4°C. The upper hexane phase, containing the lipids, was transferred to a new Eppendorf tube, hexane extraction was repeated twice. Finally, hexane was evaporated using Speed Vac Vacuum concentrators and the lipid extract was injected into a high-performance liquid chromatography (HPLC) system to determine CoQ content as previously described (Rodríguez-Aguilera et al., 2017).

Plasma was obtained from the venous plexus blood and acyl-carnitine composition was analyzed by HPLC, electrospray ionization and tandem mass spectrometry in the underivatized form, using a commercial kit or deuterated acyl-carnitines (Perkin Elmer, Madrid, Spain). β-hydroxybutyrate plasma levels were measured using an automated spectrophotometric method (Artuch et al., 1995) while free fatty acids levels in plasma were determined with an ABX Pentra 400 commercial kit NEFA C (Z52550, FUJIFILM Wako Chemicals Europe GmbH, Neuss, Germany).

Non-targeted metabolomics studies were performed on mouse liver, skeletal muscle, and plasma (stored at −80°C until processed) by the University of California Davis, West Coast Metabolomics Center (Davis, CA, United States). Analyses were performed using Gas chromatography coupled to mass spectrometry (GC-MS) methods as previously described (Mitchell et al., 2016).

### Western Blotting and Immunodetection

Skeletal muscle (500 mg) was homogenized through sonication in RIPA buffer [TrisHCl 50 mM pH = 7.4, NaCl 150mM, EDTA 1mM, 1% (v/v) NP40, Na_3_VO_4_ 5mM, Na₄P₂O₇, 10mM, protease inhibitor cocktail (PIC) 1X]. Lysate was then centrifuged to eliminate debris and unhomogenized tissue, and supernatant transferred to a new tube. After protein quantification, 40 µg were mixed with LB 1X [10% (w/v) SDS, dithiothreitol (DTT) 500 mM, 50% (v/v) Glycerol, Tris-HCL 250 mM and 0.5% (w/v) bromophenol blue dye, pH6.8.] with dithiothreitol (200 mM), loaded in a polyacrylamide gel (TGX Stain-Free FastCast Acrylamide Kit, 7.5%, 10% and 12%. Bio-Rad, Hercules, CA, United States) and subjected to electrophoresis under denaturing conditions (SDS-Page), for 40 min at 200 V using a Mini-PROTEAN Tetra cell (Bio-Rad, Hercules, CA, United States) with 1X Tris/Glycine/SDS (TGS) buffer. After electrophoresis, the gel was activated by ultraviolet (UV) exposure using a Bio-Rad ChemiDoc imager, and Stain-Free gel image was used as loading control (Gilda and Gomes, 2013) (Supplementary Figure S1). The gel was blotted onto a 0.2 µm nitrocellulose membrane, t using a Trans-Blot Turbo Transfer System (Bio-Rad). The membrane was blocked with 5% milk (blotting-Grade Blocker; BioRad Hercules, CA, United States) in Tween-Tris-buffered saline (TTBS) [20 mM (w/v) Tris-HCL, 0.15 M (w/v) NaCl, 0.5% (v/v) Tween20, pH = 7.4] for 1 h at room temperature (RT), and incubated overnight with the specific primary antibody (Supplementary Table S1) diluted in blocking solution. The membrane was washed three times in TTBS and incubated for 2 h at RT with the appropriate secondary antibody (Supplementary Table S2) conjugated with horseradish peroxidase (HRP). Finally, the membrane was incubated with Crescendo Western HRP substrate (Milipore Burlington, MA, United States) and examined using a ChemiDoc TM XRS + Imaging System (BioRad, Hercules, CA, United States). Band intensity was determined with Image Lab Software (BioRad, Hercules, CA, United States).

### Quantitative Real-Time PCR

Quantitative real-time PCR (qPCR) was used to analyze the expression of specific target genes in skeletal muscle. The HPRT housekeeping gene was used as normalization control. RNA from gastrocnemius muscle was isolated with Trizol (Invitrogen, Carlsbad, CA, United States), following manufacturer’s instructions. DNase-treated RNA (1 µg) was retrotranscribed with Iscript cDNA synthesis Kit (Bio-Rad, Hercules, CA, United States) to produce complementary DNA (cDNA).

qPCR was performed using a CFX Connect Real-Time PCR Detection System (Bio-Rad, Hercules, CA, United States) with iTaq universal SYBR Green Supermix kit (Bio-Rad, Hercules, CA, United States) in 96 wells reaction plates according to manufactured instructions. Primers sequences can be found in Supplementary Table S3.

### Skeletal Muscle Histology and Immunochemistry

The tibialis anterior (TA) muscle was carefully dissected, embedded in Tissue-Tec® OCT™ (Sakura, Torrance, CA, United States) and immediately frozen in liquid nitrogen-cooled isopentane. Cryosections of TA muscle (12 μm) were obtained with a cryostat (Leica CM1850 UV-1-1; Leica, Wetzler, Germany) and stored at −80°C until needed. For immunochemistry, slides were equilibrated at RT for 5 min and transferred to a humidified slide chamber. Slides were washed with PBS and incubated for 1 h with blocking solution [10% (v/v) fetal bovine serum, (FBS) in PBS]. Next, a cocktail of primary antibodies (described in Supplementary Table S1) diluted in blocking solution was prepared and incubated for 1 h at RT, as previosuly described (Bloemberg and Quadrilatero, 2012). Slides were then washed with PBS 3 times for 5 min, and incubated with secondary antibodies (Supplementary Table S2) for 1 h in darkness. Slides were washed 3 times for 5 min with PBS, and mounted using glycerol 50% (V/V) under a coverslip. All steps were performed in a humidified chamber. Image acquisition was performed using a Stellaris Confocal Laser Scanning Microscope (Leica, Wetzler, Germany) (10X objective); complete reconstruction of the transversal section of the TA muscle was automatically generated by Leica software. Then a section of 2000 μm × 2000 μm, for each sample, including red and white areas, from the TA muscles were used for comparative analysis, which was performed with Fiji (ImageJ) by quantifying the number of the different types of myofibers.

### Primary Satellite Cell Culture From Mouse Skeletal Muscle

Extensor digitorum longus (EDL) muscles were carefully dissected, and enzymatically digested with collagenase Type I (Sigma-Aldrich, Spruce St, MO, United States) 0.2% (w/v) in DMEM + Glutamax medium (Gibco, Waltham, MA, United States) for 2 hours at 37°C. Once muscles looked less defined and slightly swollen, they were transferred to a Petri dish with DMEM inside a sterile culture hood and triturated repeatedly to produce single, hairlike myofibers, using a heat-polished Pasteur pipette precoated with BSA 5% (w/v) in PBS as previously described (Moyle and Zammit, 2014). Clean myofibers were separated from hypercontracted myofibers, fat droplets, tendon and debris, and transferred to proliferation medium [59% [v/v] DMEM + Glutamax (Gibco, Waltham, MA, United States), 30% (v/v) FBS (Gibco, Waltham, MA, United States), 10% (v/v) horse serum (HS) (Gibco, Waltham, MA, United States), 1% (v/v) chicken embryo extract (CEE) (ThermoFisher, Waltham, MA, United States), 0.0001% (v/v) fibroblast growth factor (FGF) from (Prepotech, Rocky Hill, NJ, United States), 0.001% (v/v) penicilim-streptomycin (PS) (Sigma-Aldrich, Spruce St, MO, United States)] in a new Petri dish precoated with Matrigel (Sigma-Aldrich, Spruce St, MO, United States) [1:10 (v/v) in [DMEM + Glutamax, Gibco, Waltham, MA, United States]. Myofibers were cultured at 37°C and 5% CO_2_ for 72 h to allow satellite cell activation and migration. Under a stereo dissecting microscope placed inside a culture hood, attached myofibers were removed with a 1 ml pipette by gently agitation of the medium over the myofibers. Cells were washed twice with PBS, trypsinized for 5 min at 37°C [1 × 0.25% (w/v) trypsin-EDTA (Gibco, Waltham, MA, United States)], transferred to a non-coated 10 cm Petri dish containing fresh medium (composition mentions above) and incubated at 37°C and 5% CO_2_ for 15 min to remove contaminating fibroblasts, attached to the dish. Non-adhered satellite cells were recovered with the medium and plated on Matrigel-coated plates as described above (Moyle and Zammit, 2014). Finally, satellite cells were amplified in proliferation medium and plated in differentiation medium [97.9% (v/v) DMEM + Glutamax (Gibco, Waltham, MA, United States], 0.001% PS (Sigma-Aldrich, Spruce St, MO, United States) and 2% mouse blood serum). For serum extraction, blood samples were collected every 2 weeks from mice of both genotypes on *ad libitum* and CR regimes at 08:30 a.m., and incubated for 1.5 h at RT in an Eppendorf. Coagulated blood was centrifuged at 2000 g for 15 min at RT, serum collected from the upper phase, and filtered through 0.45 µm pore size filter before use.

### Immunostaining of Satellite Cells

Satellite cells were plated in a 96-well plate previously coated with Matrigel [1:10 (v/v) in DMEM + Glutamax [Gibco, Waltham, MA, United States)] for the assays. Cells were fixed with 100 µl of 4% (w/v) paraformaldehyde per well, and the plate was transferred to an orbital shaker at RT for 8 min. Cells were then washed 3 times with PBS for 5 min, and permeabilized with 0.1% (v/v) Triton/PBS for 15 min on an orbital shaker at RT. Cells were incubated with blocking solution (5% [v/v] FBS/PBS) for 1 h an orbital shaker at RT. Myosin Heavy Chain (MHC) antibody diluted in blocking solution (1:100) (Supplementary Table S1) was incubated overnight on an orbital shaker at 4°C. Cells were washed 3 times with PBS for 5 min and secondary antibody (Supplementary Table S2) was incubated for 2 h on an orbital shaker at RT in darkness. Cells were washed with PBS and incubated with DAPI (Sigma-Aldrich, Spruce St, MO, United States) (1 μg/ml) for 10 min on an orbital shaker at RT. Images were acquired on a Fluorescence Microscopy Zeiss Axio Imager M2 (Carl Zeiss Meditec AG, Oberkochen, Germany) (10X objective). MHC area was calculated using a high throughput image analysis R script (Banerji et al., 2019). Five images per well were used to calculate the mean MHC area and five wells were used for each cell line and time point. Myotubes length and branching capacity were studied with Fiji software.

### Assessment of Mitochondrial Respiration in Satellite Cell Derived Myotubes

Agilent Seahorse XFe24 (Agilent, Santa Clara, CA, United States) microplate-based oxygen consumption technology was used to analyze respiration in myotubes differentiated from satellite cells. Previously, the XFe24 microplate was precoated with Matrigel [1:10 (v/v) in DMEM + Glutamax]. Then, freshly isolated satellite cells were seeded at an optical density of 30.000 cells onto Matrigel pre-coated well as described above, and cultured with differentiation medium [97.9% (v/v) DMEM + Glutamax (Gibco, Waltham, MA, United States), 0.001% (v/v) PS (Sigma-Aldrich, Spruce St, MO, United States), 2% serum isolated from mice) for 72 h. oxygen consumption rate (OCR) was measured in Agilent Seahorse XF base medium supplemented with 2 mM D-glucose, 1 mM sodium pyruvate and 2 mM l-glutamine and pH was adjusted to 7.4. Substrates and inhibitors were sequentially added as follows: oligomycin (4 μM final), carbonyl cyanide-p-trifluoromethoxy phenylhydrazone (FCCP) (1.0 μM final), antimycin A (5 μM final) and rotenone (1 μM final). Respiratory parameters for OCR assay were calculated as previously described (Divakaruni et al., 2014). The β-oxidation assay was performed in MAS buffer [sucrose (70 mM), mannitol (440 mM), KH_2_PO_4_ (100 mM), MgCl_2_ (50 mM), EGTA (10 mM), and HEPES (20 mM)]; substrates and inhibitors used were digitonin plus malate (5 μg/μl and 2.5 mM final, respectively), palmitoyl-DL-carnitine plus ADP (50 μM and 1 mM final, respectively), oligomycin (4 µM final) and antimycin A plus rotenone (5 µM final and 1 µM final, respectively). Malate was added to facilitate the metabolization of extra acetyl-CoA generated in fatty acids β-oxidation. Fatty acids β-oxidation rates were obtained subtracting the two measures post digitonin/malate injection from the two measures post ADP/palmotoyl-DL-carnitine. After measurements were obtained, cells were lysed in RIPA buffer for protein quantification. RC DC Protein Assay kit (Bio-Rad 5000122) was used to quantify protein content and albumin (Bio-Rad 5000007) was used as standard for normalization. Citrate synthase activity was determined in skeletal muscle homogenates by spectrophotometric assays as previously described (Spinazzi et al., 2012).

### Assessment of Respiration in Isolated Mitochondria From Skeletal Muscle

To assess mitochondrial metabolism and bioenergetics, mitochondria were isolated from skeletal muscle through serial centrifugations, 10 µg of mitochondrial fraction was seeded per well as previously described (Hernández-Camacho et al., 2022). Assays were conducted using an Agilent Seahorse XFe24 microplate-based oxygen consumption system. The Coupling Assay (CA) examines mitochondrial states in the presence of pyruvate and malate as substrates. The β-oxidation of fatty acids assay (BOX) was carried out in the presence of palmitoyl-l-carnitine and malate. Substrates and inhibitors were sequentially added as follows: ADP (5 mM final) for CA and (4 mM final) for BOX, Oligomycin (3.16 μM final), FCCP (6 μM final) and antimycin A plus rotenone [4 and 5 μM final, respectively) for CA or antimycin A plus rotenone (4 and 2 μM final, respectively) for BOX.

### Statistics

Data were expressed as mean ± SD. Data analysis were performed with GraphPad Prism 8.0 software. When a comparison between two groups was performed, an unpaired two-tailed *t*-test with Welch’s correction was used. When more than two groups were studied, a one way (1 parameter studied) or two-way ANOVA (2 or more parameters studied) with Sidak’s, Tukey’s or Dunnett’s multiple comparison post-hoc test was applied. *p*-values < 0.05 were considered statistically significant.

## Results

### CR Restores Metabolic Homeostasis in Adck2^+/−^ Mice

**FIGURE 1:**
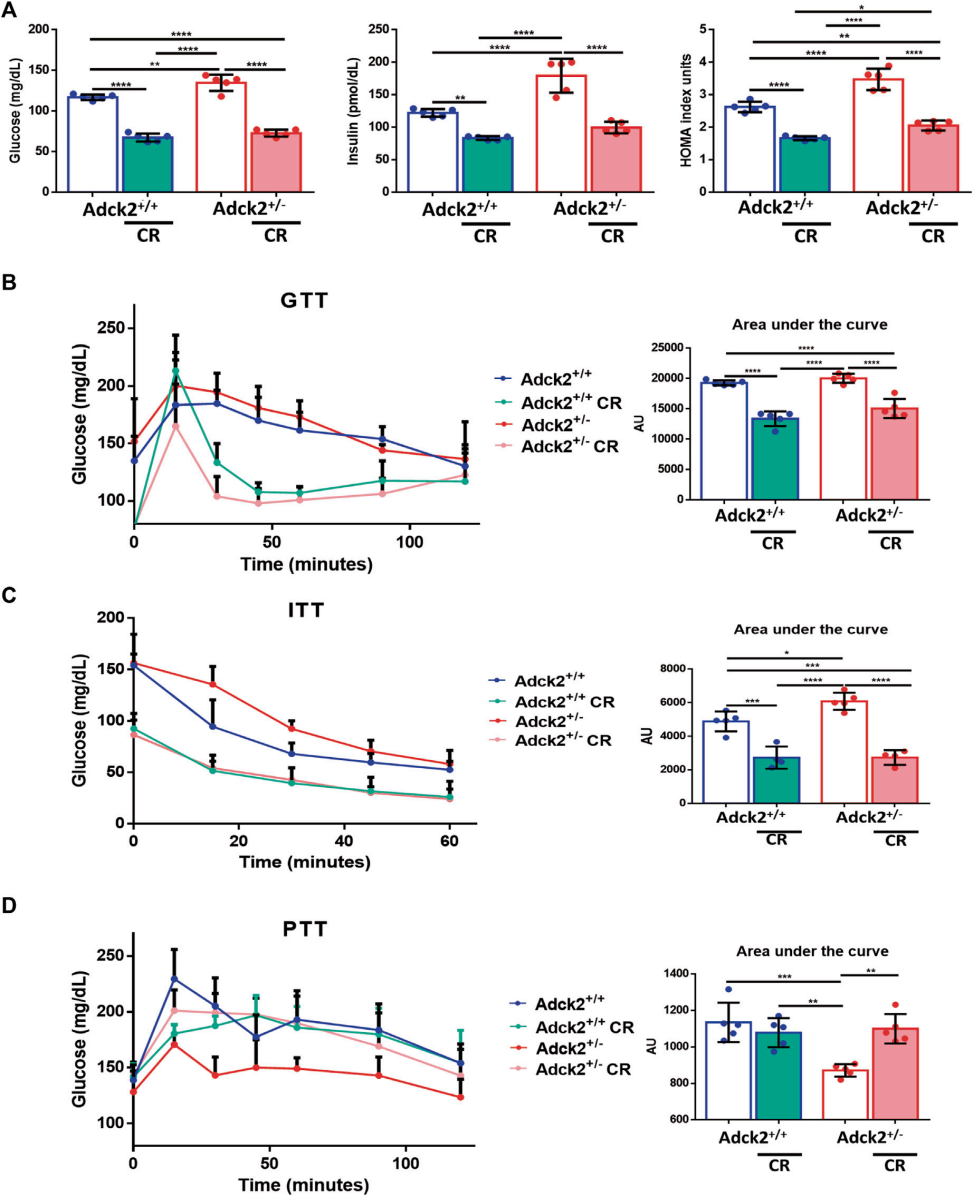
Glucose and insulin homeostasis on calorie restriction conditions. **(A)**. Fasting glucose and insulin levels in plasma, HOMA index (*N* = 5). **(B)**. Glucose tolerance test (GTT), glucose concentration in blood after glucose load and area under the curve (AUC) quantification (N = 5). **(C)**. Insulin tolerance test (ITT), plasma levels of glucose after insulin injection and AUC quantification (N = 5). **(D)**. Pyruvate tolerance test (PTT). Glucose levels in blood after pyruvate injection and AUC quantification (N = 5). CR, calorie restriction. Data represent the mean ± SD. One-way ANOVA test was applied. *p < 0.05; **p < 0.01; ***p < 0.001; ****p < 0.0001.

To evaluate if CR could be beneficial for mitochondrial dysfunction treatment, six-month-old male *Adck2* mutants (*Adck2* ^+/−^) mice were placed on a 40% CR for 7 months, and tested against age-matched wild type (WT) controls. A second control group consisted of wild type and mutant mice of the same age on *ad libitum* feeding conditions. *Adck2* ^+/−^ mice on *ad libitum* diet showed a higher weight gain compared with WT mice, while no differences were observed between these two genotypes when on CR (Supplementary Figure S1B). Due to the implications of CR on glucose homeostasis, fasting levels of glucose and insulin were determined (Figure 1A). Both parameters were higher in *Adck2* ^+/−^ on *ad libitum* diet compared with WT, while both groups of mice on CR showed an important reduction in glucose and insulin levels. The HOMA index was higher on *Adck2* ^+/−^ mice on *ad libitum* diet compared with WT, while CR decreased HOMA index in both groups. Glucose tolerance test (GTT) had a tendency to exhibit higher area under the curve in *Adck2* ^+/−^ mice on *ad libitum* diet compared with WT, and CR induced lower levels of glucose in both genotypes (Figure 1B). Similar results were obtained with the insulin tolerance test (ITT), in which *Adck2* ^+/−^ mice subjected to CR showed a faster decrease of glucose in plasma in comparison with *ad libitum* fed animals, suggesting an improvement of glucose entry into the cell by increasing insulin sensitivity (Figure 1C). Hepatic gluconeogenesis was evaluated through the pyruvate tolerance test (PTT), showing a lower glucose production by *Adck2* ^+/−^ in comparison with WT mice fed with *ad libitum* diet. *Adck2* ^+/−^ mice on CR showed a rise in glucose levels after pyruvate injection (Figure 1D). Altogether, these results indicated that *Adck2* ^+/−^ mice on an *ad libitum* diet exhibit higher insulin resistance and impaired glucose homeostasis, but when fed under CR conditions, insulin resistance is reverted.

**FIGURE 2:**
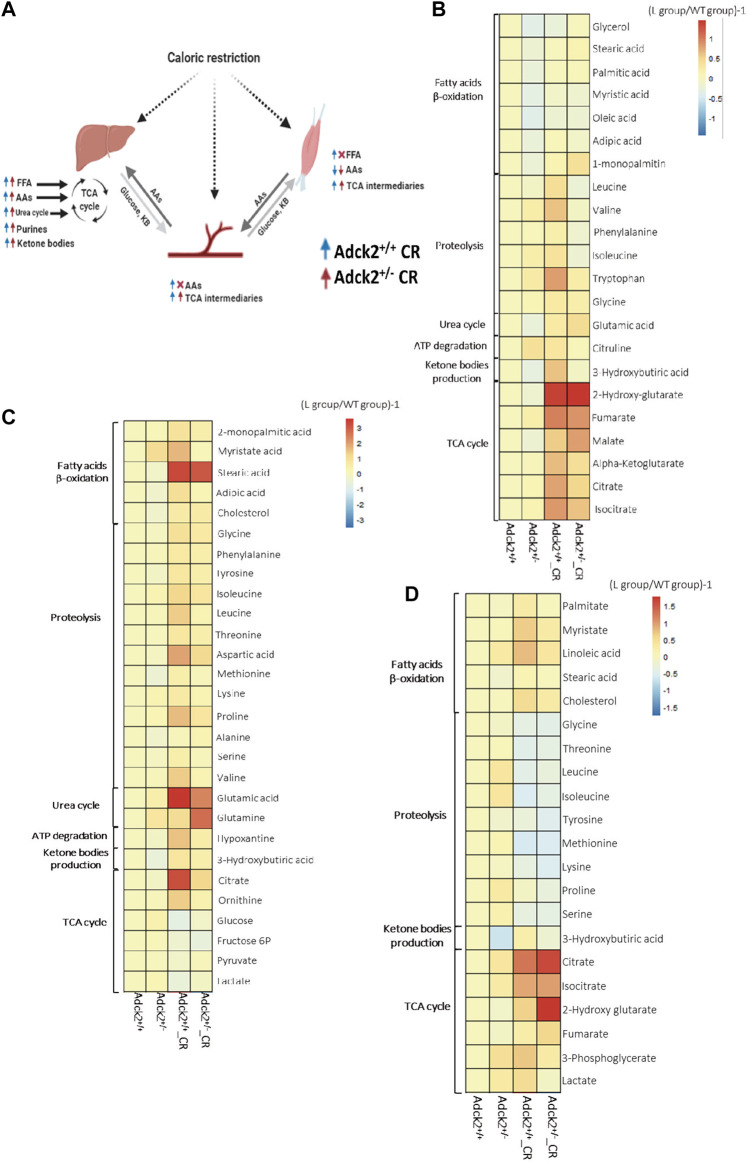
Metabolic profile in plasma, liver and muscle on calorie restriction conditions. **(A)**. Summary of the effects of calorie restriction on metabolomics. **(B)**. Metabolic profile in the plasma. (Adck2^+/+^ N = 3, Adck2^+/−^ N = 3, Adck2^+/+^ CR N = 4, Adck2^+/−^ CR N = 4). **(C)**. Metabolic profile in liver. (Adck2^+/+^ N = 4, Adck2^+/−^ N = 4, Adck2^+/+^ CR N = 5, Adck2^+/−^ CR N = 5). **(D)**. Metabolic profile in skeletal muscle. (Adck2^+/+^ N = 4, Adck2^+/−^ N = 4, Adck2^+/+^ CR N = 5, Adck2^+/−^ CR N = 5). Statistics analyses are included in supplementary materials. CR, calorie restriction; FFA, free fatty acids; AAs, aminoacids; TCA, tricarboxylic cycle.

To understand further the effect of CR on *Adck2* ^+/−^ metabolism, a non-targeted metabolomics analysis was performed using three different mouse samples: plasma, skeletal muscle, and liver, key metabolites from different bioenergetics pathways are depicted in the Figure 2. The liver is the main modulator of energy substrate metabolism in terms of oxidative and glycolytic pathways. Plasma is the main indicator of the metabolic responses to CR in peripheral tissues. The main tissue affected in human patients was skeletal muscle (Vázquez-Fonseca et al., 2019), which is also highly responsive to CR. We found that CR modulated liver metabolism similarly in WT and *Adck2* ^+/−^ mice compared with *ad libitum* fed mice (Figure 2C). The livers of CR mice were adapted to use energy from peripheral tissues for subsequently gluconeogenesis, including increased levels of fatty acids and amino acids (essential, gluconeogenic and ketogenic amino acids), and an elevation of metabolites of bioenergetic pathways (TCA cycle, urea cycle and purines biosynthetic pathway). Plasma metabolic profiles (Figure 2B) showed an elevation of TCA cycle metabolites and amino acids in WT animals under CR compared with *ad libitum*. However, *Adck2* ^+/−^ mice on CR did not exhibit the same increase of amino acids in plasma, probably because amino acids are mainly subjected to higher catabolic degradation in this genotype. Skeletal muscle metabolic profile in CR-WT mice showed an increase of fatty acids, amino acids, and the TCA cycle components compared with *ad libitum* diet (Figure 2D), indicating that CR promotes the use of fatty acids and amino acids as energy substrates. *Adck2* ^+/−^ mice showed an increased profile of fatty acids levels in skeletal muscle under CR but at a lower intensity than WT (Supplementary Table S4).

Metabolic profiles in the liver (Figure 2C) showed that several fatty acids were increased such as myristate, stearic acid, laureate, 2-monopalmitic acid, adipic acid and cholesterol, as well as a wide range of amino acids, including ketogenic (Phe, Tyr, Ileu) and glucogenic (Gly, Thr, Asp, Met, Gln, Glu), branched-chain (BCAAs, Val, Ileu y Leu), and essential amino acids (e.g., Val, Ileu, Leu, Met, Phe, Thr, Pro, Ala, Met). Other elevated metabolites in liver were 3-hydroxybutyrate (a ketone body) and intermediaries of the TCA cycle such as citrate and isocitrate, which together with the increment of ornithine agreed with the activation of both urea and TCA cycles. CR led to the mobilization of peripheral energy deposits that the liver could employ for gluconeogenesis. The increase in purines content in CR was probably associated with ATP degradation suggesting a more efficient energy metabolization. Mitochondria from liver on CR function mainly in catabolic mode, with fatty acid β-oxidation driving the TCA cycle. Additionally, a profile consistent with anaplerotic replenishment *via* amino acids (both ketogenic and gluconeogenic), and activation of the urea cycle was observed in both WT and *Adck2* ^+/−^ mice on CR. Under these circumstances, the anaplerotic filling of the TCA cycle, together with the energetic fatty acid fueling of mitochondria rendered a metabolism relying less on glucose, because the levels of intermediaries of the glycolytic pathway were decreased. In response to CR, liver metabolic profile from WT and *Adck2* ^+/−^ mice showed an increase in fatty acids use as a fuel to provide energy to the rest of energy-dependent tissues, suggesting a shift in hepatic substrate utilization toward gluconeogenesis instead of glycolysis.

Metabolites found in CR-plasma (Figure 2B) suggested an induction of catabolism and an accumulation of metabolites for the TCA cycle and fatty acids as previously reported (Guijas et al., 2020) indicating an adaptation to food deprivation and a switch to a metabolic mode in which fatty acids are used as energetic substrate (Longo et al., 2021). An accumulation of several amino acids in plasma of CR mice was also identified, including ketogenic (e.g., Ileu) and gluconeogenic (e.g., Gly), branched-chain (BCAAs, Val, Ileu, Leu) and essential (e.g., Phe and Trp) amino acids. TCA cycle metabolites and fatty acids were also elevated in the plasma of mutant mice on CR, suggesting a mobilization of peripheral energy deposits. Because of the catabolic metabolism adapted in the liver of mice on CR, the TCA cycle metabolites were increased in plasma to feed the different tissues of the organism of the mice on CR. Additionally, CR induced activation of purines and metabolites from the urea cycle.

Metabolic profiles from skeletal muscle (Figure 2D) revealed that CR induced metabolic adaptations associated with energy deprivation. In WT mice, CR induced an increase in fatty acids levels and TCA metabolites, and a decrease in amino acids. *Adck2* ^+/−^ on CR showed a lower elevation of fatty acids in the muscle when compared to mutant mice on *ad libitum* diet. Both sets of data are indicative of a metabolic energy substrate switch from glucose to fatty acids, which was confirmed by the accumulation of fumarate, the final product of the mitochondrial complex II reaction (Spinelli et al., 2021). Therefore, CR intervention in *Adck2* ^+/−^ mice showed a metabolic switch to increase the use of fatty acids instead of glucose as energy substrate.

### CR Improves Physical Activity in Adck2^+/−^ Mice

**FIGURE 3:**
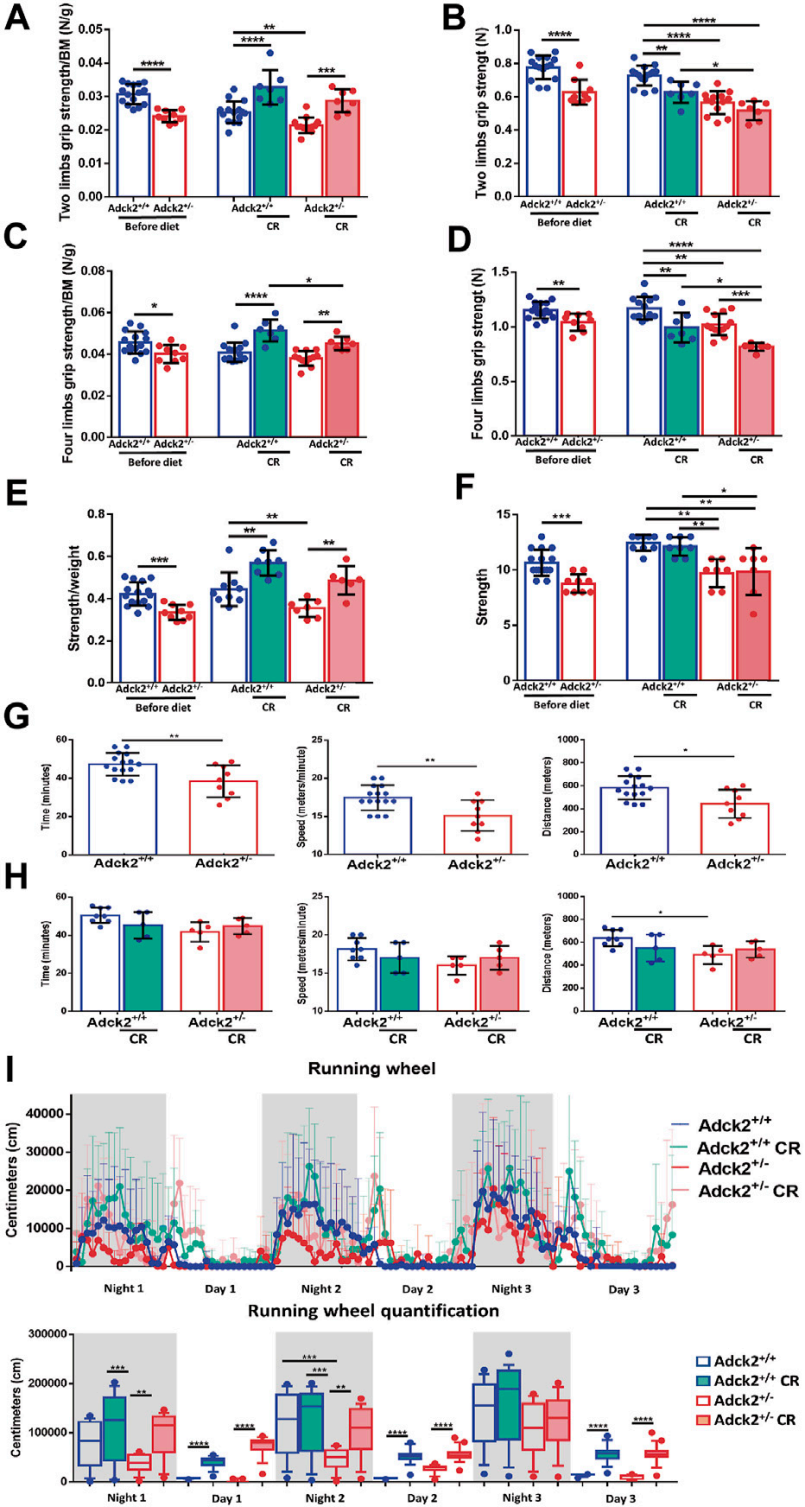
Calorie restriction adaptations on physical performance. Results for two and four limbs grip strength tests, weight-lifting normalized by body weight and aerobic treadmill tests are shown on basal conditions before starting and at end of diet periods **(A,C,E,G,H)**. Two and four limbs grip strength tests and weight-lifting test without normalization **(B,D,F)**. **(I)** Voluntary wheel running activity in the automated home cage phenotyping system. Data are expressed as distance recorded in centimeters (cm). CR, calorie restriction; BM, body mass. In all cases, data represent the mean ± SD. A, B, C y D (before diet Adck2^+/+^ N = 15, Adck2^+/−^ N = 9. After diet Adck2^+/+^ N = 15, Adck2^+/−^ N = 13 Adck2^+/+^ CR N = 7, Adck2^+/−^ CR N = 7). E and F (before diet Adck2^+/+^ N = 15, Adck2 N^+/−^ = 9. After diet Adck2^+/+^ N = 9, Adck2^+/−^ N = 7 Adck2^+/+^ CR N = 8, Adck2^+/−^ CR N = 7). G (Adck2^+/+^ N = 16, Adck2^+/−^ N = 9). H (Adck2^+/+^ N = 8, Adck2^+/−^ N = 5 Adck2^+/+^ CR N = 5, Adck2^+/−^ CR N = 5). I (Adck2^+/+^ N = 7, Adck2^+/−^, N = 5 Adck2^+/+^ CR N = 7, Adck2^+/−^ CR N = 8). One-way ANOVA test was applied. *p < 0.05; **p < 0.01; ***p < 0.001; ****p < 0.0001. Regression-based analysis of absolute strength using body mass as covariate (ANCOVA) was also applied to the strength tests without normalization by body mass **(B,D,F)** giving the following *p*-values: 0,0000025 (B, two limbs grip strength test), 0,0045 (D, four limbs grip strength test) and 0,00042 (F, weight-lifting test).

To determine if the partial metabolic reprograming of skeletal muscle in *Adck2* ^+/−^ induced by CR was enough to improve physical performance, physical capacity was analyzed (Figure 3). Strength was evaluated with two and four limb grip strength tests, and a weight lifting test. Before starting CR, *Adck2* ^+/−^ mice exhibited lower strength than WT animals in all tests (Figures 3 A,C,E). WT and *Adck2* ^+/−^ mice showed an increase in strength/per unit of mass for both tests after 7 months on CR (Figures 3 A,C,E), suggesting that CR could improve *in vivo* strength/per unit of mass in both genotypes.

Oxidative metabolism dependent pathways were determined by long-term and low-speed aerobic forced running capacity treadmill tests. Before CR, *Adck2* ^+/−^ mice ran for less time and at a lower speed and covered a shorter distance compared to WT mice (Figure 3G); *Adck2* ^+/−^ mice fed on CR display a tendency to increase running capacity in the three parameters analyzed (Figure 3H). Additionally, voluntary running wheel capacity was also examined over 3 days in a TSE PhenoMaster system. *Adck2* ^+/−^ mice on an *ad libitum* diet exhibited a lower accumulated distance compared to WT mice, but both genotypes on CR conditions considerably increased the freely running distance, reinforcing CR as an enhancer of aerobic capacity in skeletal muscle (Figure 3I).

### CR Increases Oxidative Myofibers in Adck2^+/−^ Skeletal Muscle

**FIGURE 4:**
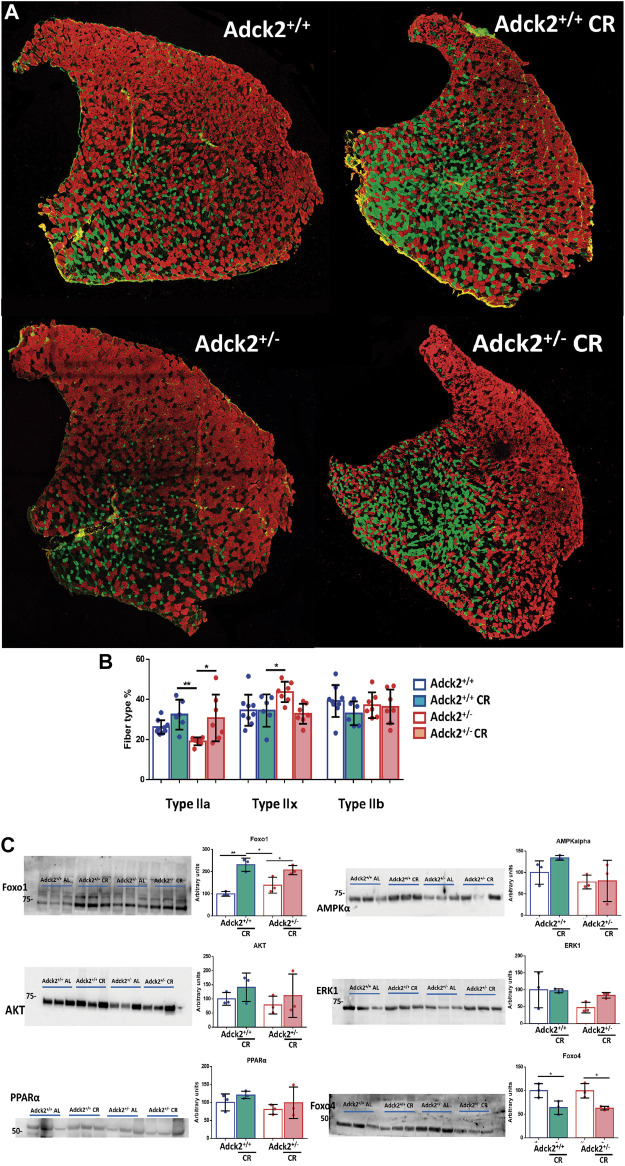
Myofiber composition in skeletal muscle under calorie restriction conditions. **(A)**. Representative images of tibialis anterior (TA) muscles from 13 month-old mice. Type IIa, IIb, and IIx myofibers appear in green, red and black, respectively. **(B)**. Myofiber composition analysis. Data represent the mean ± SD (Adck2^+/+^ N = 9, Adck2^+/−^ N = 7, Adck2^+/+^ CR N = 6, Adck2^+/−^ CR N = 7) **(C)**. Protein levels of metabolic markers performed in protein homogenates from gastrocnemius muscles. CR, calorie restriction. Data represents the mean ± SD (N = 3). Unpaired two-tailed *t*-test and one-way ANOVA test were applied. *p < 0.05; **p < 0.01; ***p < 0.001; ****p < 0.0001.

Based on the results of the reprogrammed metabolism and physical capacity improvement promoted by CR, we hypothesized that skeletal muscle fiber type change could explain the effects on physical activity. *Adck2* ^+/−^ mice on an *ad libitum* diet showed a lower proportion of type IIa myofibers and an increase in type IIx myofibers compared to WT (Figures 4A,B), indicating an altered myofiber composition. CR produced an increase in type IIa myofibers in both genotypes (Figure 4A) inducing a more oxidative myofiber profile for both groups of mice (Figure 4B).

In parallel, protein levels of metabolic factors regulated by CR were examined in skeletal muscle (Figure 4C). FOXO1 and AMPKα, two proteins that form part of food deprivation activated pathway, were upregulated in both genotypes fed under CR (Hofer et al., 2022). PPARα was also found to be activated in both genotypes on CR. The increase of AKT in mice fed under CR could indicate a higher sensibility to insulin, while the response observed in ERK levels on mice from CR groups could indicate activation of apoptosis. CR intervention induced lower levels of FOXO4 in skeletal muscle, which probably contributes to the observed effect on glucose homeostasis. These changes evidenced acclimatization to reduced energy intake and fasting in skeletal muscle under CR.

### CR Improved Aerobic Respiration in Adck2^+/−^ Skeletal Muscle

**FIGURE 5:**
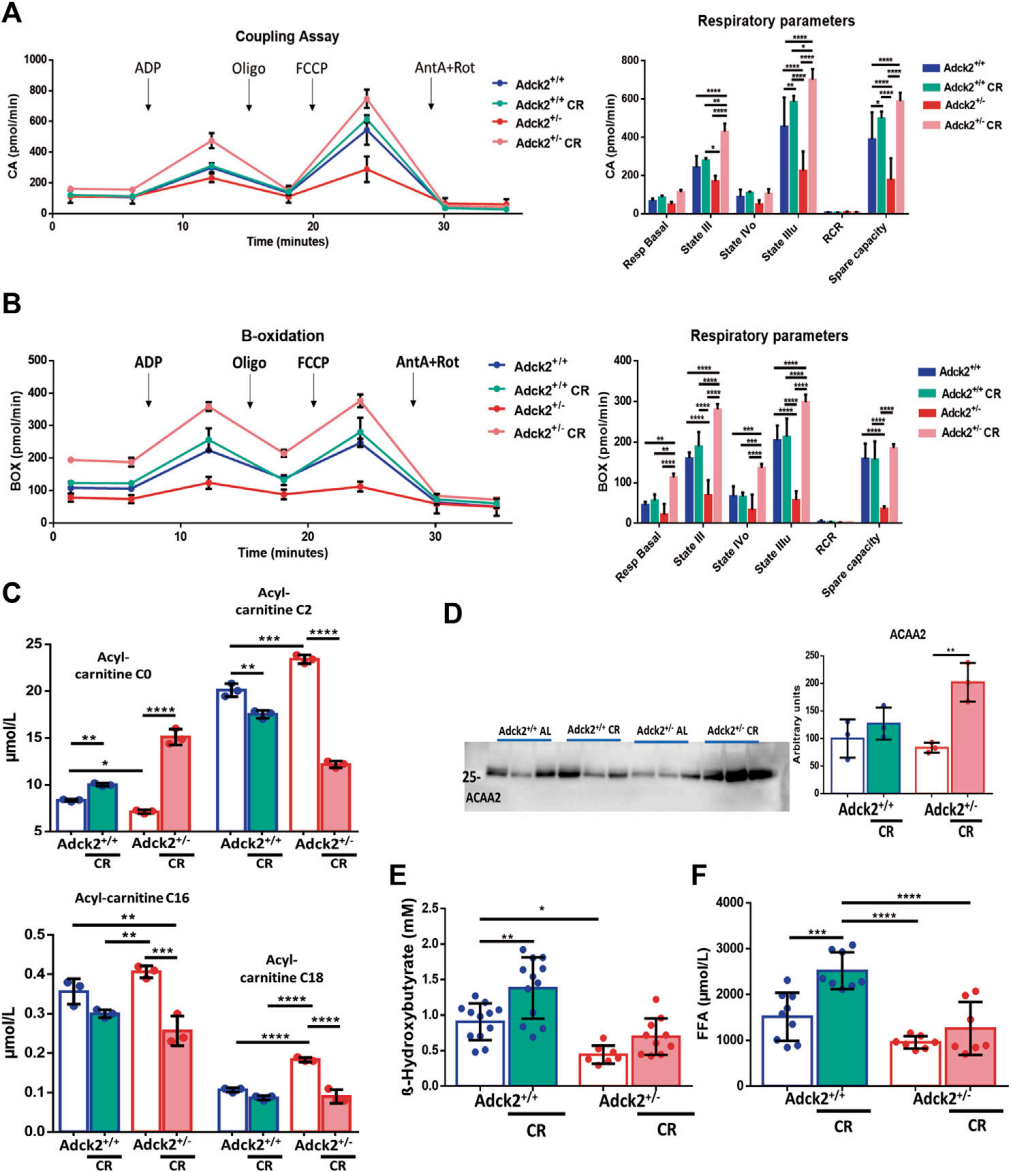
Bioenergetics approach to calorie restriction and mitochondria metabolism. **(A)**. Coupling Assay and **(B)** Fatty acids β-oxidation on mitochondria isolated from skeletal muscle on calorie restriction. For each experiment 10 µg of mitochondrial suspension was seeded per well. One representative experiment of three independent experiments in shown. RCR for Coupling assay: *Adck2* ^+/+^ 5.30 ± 1.39, *Adck2* ^+/+^ CR 5.28 ± 0.13, *Adck2* ^+/−^ 5.30 ± 1.59, *Adck2* ^+/−^ CR 6.93 ± 1.28. RCR for β-oxidation assay: *Adck2* ^+/+^ 2.39 ± 0.08, *Adck2* ^+/+^ CR 3.24 ± 0.26, *Adck2^+/−^* 1.35 ± 0.34, *Adck2* ^+/−^ CR 2.19 ± 0.06. **(C)**. Acyl-carnitine (C0), (C2), (C16), (C18) measurements in plasma. Data represent the mean ± SD (N = 3). **(D)** Acetyl-CoA Acyltransferase 2 (ACAA2) protein levels in skeletal muscle. Data represent the mean ± SD (N = 3). **(E)**. β-Hydroxybutyrate plasma levels. Data represent the mean ± SD (Adck2^+/+^ N = 12, Adck2^+/−^ N = 9, Adck2^+/+^ CR N = 12, Adck2^+/−^ CR N = 11). **(F)**. Free fatty acid plasma levels. Data represent the mean ± SD (Adck2^+/+^ N = 9, Adck2^+/−^ N = 7, Adck2^+/+^ CR N = 8, Adck2^+/−^ CR N = 7). One-way and two-way ANOVA tests were applied. CR, calorie restriction. *p < 0.05; **p < 0.01; ***p < 0.001; ****p < 0.0001.

To further characterize the impact on energy substrate utilization driven by the metabolic changes identified, We performed respirometry analysis on isolated skeletal muscle mitochondria using either pyruvate/malate to stimulate the Krebs cycle and complex I directed respiration, or palmitoyl-L-carnitine/malate to stimulate fatty acids β-oxidation. Samples from *ad libitum* fed *Adck2* ^+/−^ mice showed a lower oxygen consumption in the coupling assay compared with mitochondria from WT mice, suggesting a decreased respiration capacity, especially affecting mitochondrial state IIIu and spare capacity (Figure 5A). However, samples of both genotypes under CR presented higher respiration levels than the corresponding genotype on *ad libitum* diet, indicating that CR improved mitochondrial respiration irrespectively (Figure 5A). Particularly, mitochondria from *Adck2* ^+/−^ mice on CR displayed a significant increase compared to those isolated from *Adck2* ^+/−^ mice on *ad libitum*. Furthermore, a decrease in oxygen consumption in samples from skeletal muscle of *Adck2* ^+/−^ mice compared with WT mitochondria in *ad libitum* diet was also observed in the β-oxidation assay (Figure 5B). Differences in basal respiration states III and IIIu, and spare capacity were also found. Mitochondria from mice on CR showed a significant increase in oxygen consumption, particularly samples from *Adck2* ^+/−^ mice (Figure 5B). These results support the hypothesis that CR induces a higher substrate use efficiency while activating the different bioenergetic pathways involved in mitochondrial respiration

To further explore the bioenergetics profile observed in *Adck2* ^+/−^ skeletal muscle, we analyzed the acyl-carnitine profile in plasma from the two genotypes and dietary conditions. *Adck2* ^+/−^ mice on *ad libitum* showed an accumulation of acyl-carnitines C2, C16, and C18 and the corresponding decrease of free acyl-carnitine (C0) compared to WT, indicating a defect on fatty acids transport into the cell (Figure 5C). In contrast, CR facilitated the transport of acyl-carnitines, causing a decrease in their concentration (Figure 5C). Finally, protein levels of Acetyl-CoA Acyltransferase 2 (ACAA2), which catalyzes the last step of β-oxidation, were elevated only in *Adck2* ^+/−^ mice fed under CR, indicating a higher β-oxidation capacity (Figure 5D). Additionally, both groups of mice fed with CR diet showed a 50% higher β-hydroxybutyrate plasma levels after a night of fasting, indicating a higher production of ketone bodies (Figure 5E). CR also promoted the increase of free fatty acids in plasma in both genotypes (Figure 5F).

### CR Improves Satellite Cell Differentiation *In Vitro*

**FIGURE 6:**
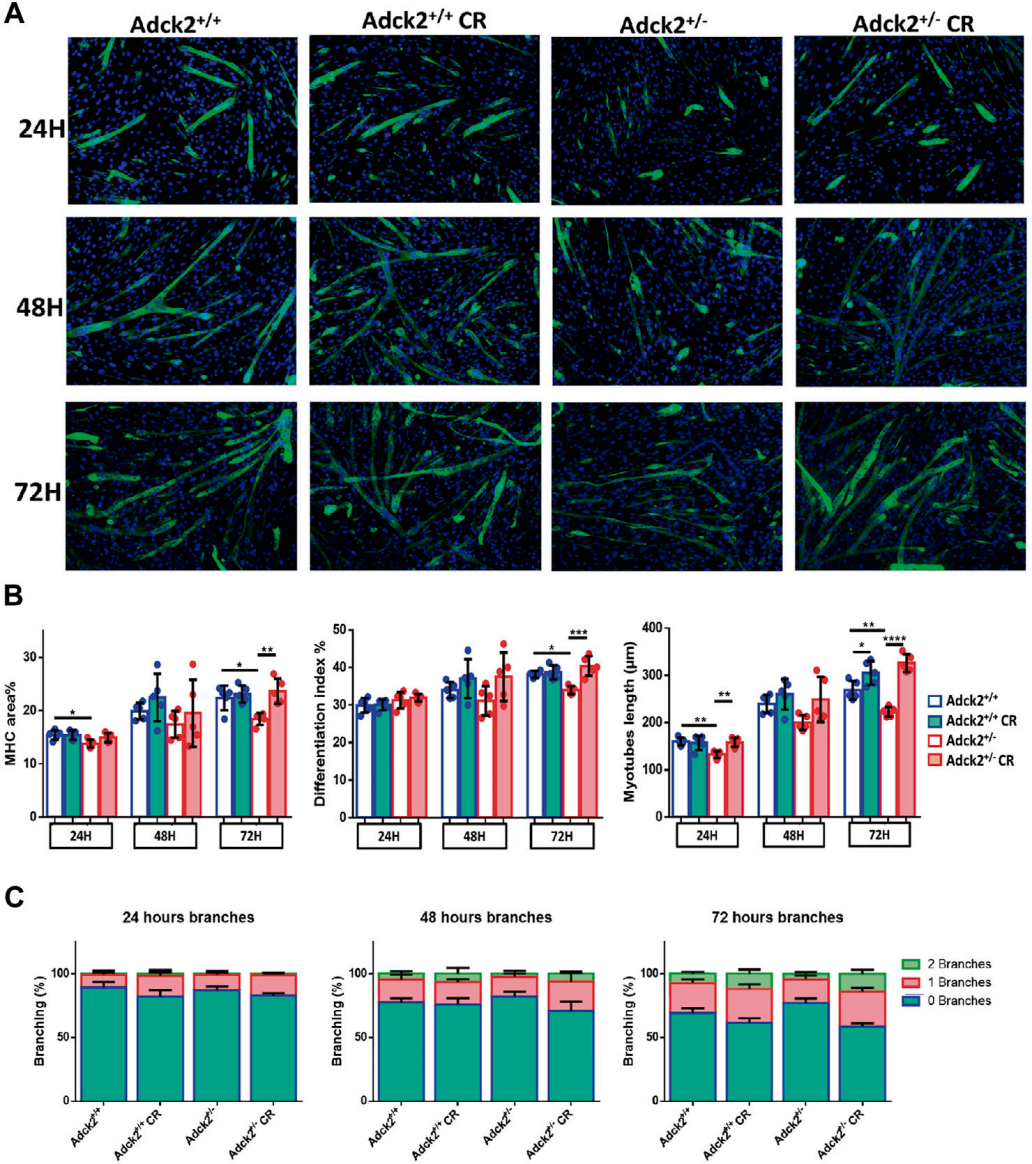
Differentiation process of satellite cells analysis under calorie restriction conditions. Development of an *in vitro* model of CR using satellite cells isolated from mice. **(A)**. Representative images of satellite cell differentiation at 24 h, at 48 h and at 72 h post-induction. **(B)** Myosin heavy chain (MHC) area, differentiation index and myotube length. **(C)**. Branching capacity during the 72 h analyzed. DAPI was used to stain the nuclei of the myotubes in blue while differentiated myotubes were stained with MHC antibody in green. The differentiation process is determined by the increase of myotube size and the MHC expression level. Differentiation of satellite cells to myotubes is induced by a reduction in serum concentration. Fresh medium was replaced in each well every 48 h. CR, calorie restriction. Data represent the mean ± SD. One-way ANOVA test was applied. *p < 0.05; **p < 0.01; ***p < 0.001; ****p < 0.0001.

**FIGURE 7:**
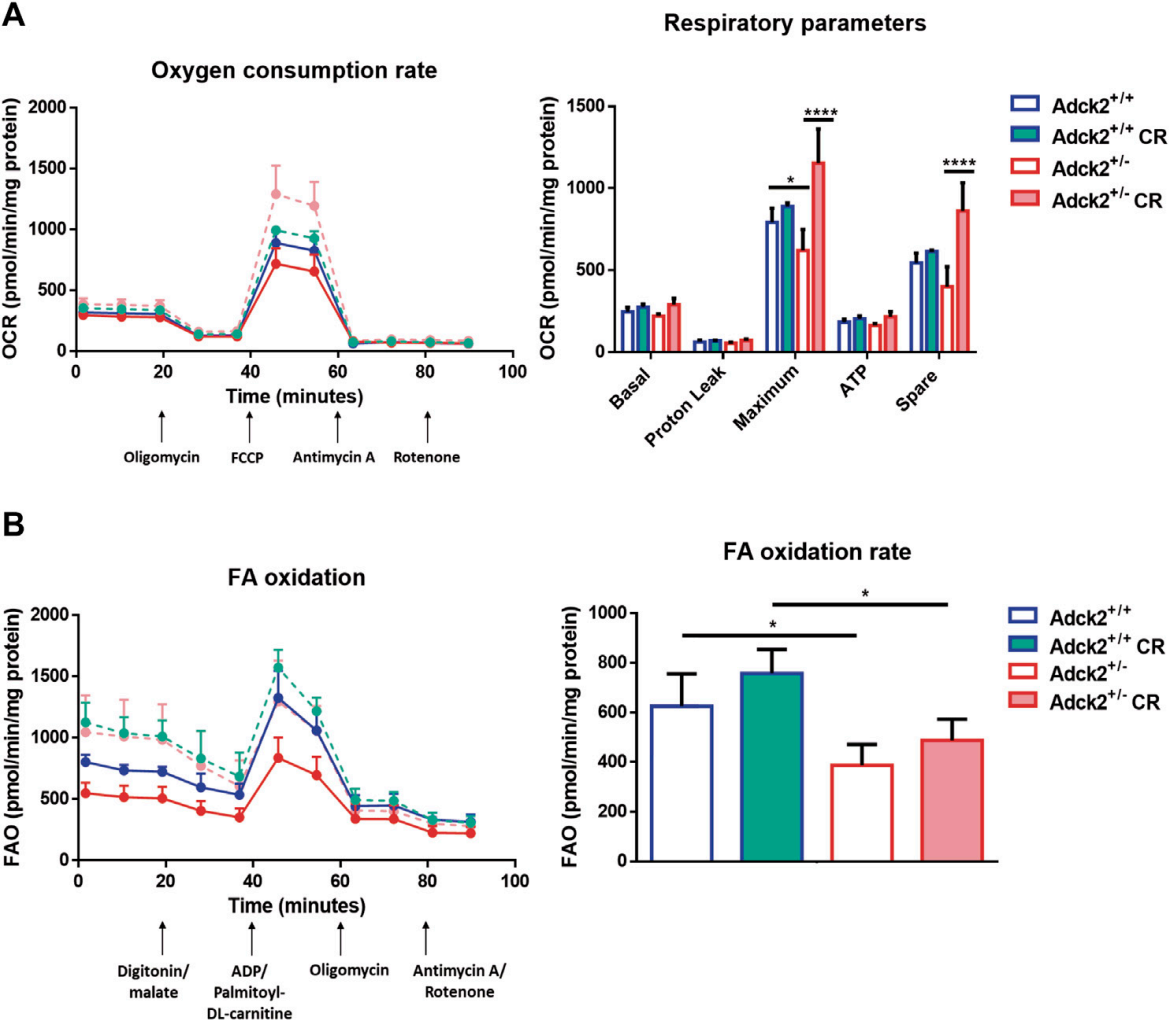
Respiration of satellite cells under calorie restriction conditions. **(A)**. Oxygen consumption rate in myotubes. **(B)**. Fatty acids β-oxidation rate in myotubes. FA β-oxidation rates were obtained subtracting the two measures post digitonin/malate injection to the two measures post ADP/palmotoyl-DL-carnitine One representative experiment of two independent experiments in shown for each assay. FA, fatty acids; FAO, fatty acids oxidation; OCR, oxygen consumption rate; FCCP, Carbonyl cyanide-p-trifluoromethoxyphenylhydrazone; CR, calorie restriction. Data represent the mean ± SD. Two-way ANOVA test was applied. *p < 0.05; **p < 0.01; ***p < 0.001; ****p < 0.0001.

Adult skeletal muscle stem cells, also known as satellite cells, are necessary for skeletal muscle remodeling, and thus we explored the possibility that the observed benefits of CR upon muscle bioenergetics and performance in *Adck2* ^+/−^ mice could be mediated by satellite cells, as previously shown in WT animals (Cerletti et al., 2012). Adult stem cells were isolated from WT and heterozygous mutant mice and cultured with serum obtained from WT and *Adck2* ^+/−^ mice both under *ad libitum* and CR conditions, with the aim to establish a microenvironment characterized by the different serum compositions. Differentiation was assessed by measuring myotube growth, Myosin Heavy Chain (MHC) area, length and branching capacity over 72 h (Figure 6A). *Adck2* ^+/−^ myotubes cultured with serum from *ad libitum Adck2* ^+/−^ mice showed lower MHC area and myotube length compared to WT myotubes cultured with serum from *ad libitum* WT mice (Figure 6B). *Adck2* ^+/−^ myotubes cultured with serum from CR *Adck2* mice showed a similar increase in MHC area, differentiation index and myotube length than WT myotubes grown with serum from CR WT mice (Figure 6B). After 72 h incubation with the different sera, *Adck2* ^+/−^ myotubes cultured with serum from on *ad libitum Adck2* ^+/−^ mice showed a lower branching capacity than WT myotubes grown in *ad libitum* derived serum. However, CR-derived serum from *Adck2* ^+/−^ induced a significant increase of branching capacity *in vitro* (Figure 6C; Supplementary Table S5). The velocity of the differentiation was also quantified through the slope of the line for each group in these parameters (Supplementary Table S6), being the differentiation velocity between 2 and 3 times greater for *Adck2* ^+/−^ satellite cells grown with CR serum compared to the same cells grown with serum from mice fed *ad libitum*. Furthermore, *Adck2* ^+/−^ myotubes cultured with on *ad libitum Adck2* ^+/−^ serum exhibited lower oxygen consumption than WT myotubes when glucose (Figure 7A), or palmitoyl-L-carnitine (Figure 7B) were used as substrates. *Adck2* ^+/−^ myotubes grown with serum from CR *Adck2* ^+/−^ mice showed increased oxygen consumption rate in respirometry assays with either glucose or fatty acids as substrates, indicating an improvement of respiration *in vitro* by the CR serum (Figures 7A,B). In summary, CR serum induced myotube differentiation *in vitro* and increased mitochondrial respiration.

### CR Improves Mitochondria Biogenesis in Adck2^+/−^ Skeletal Muscle

**FIGURE 8:**
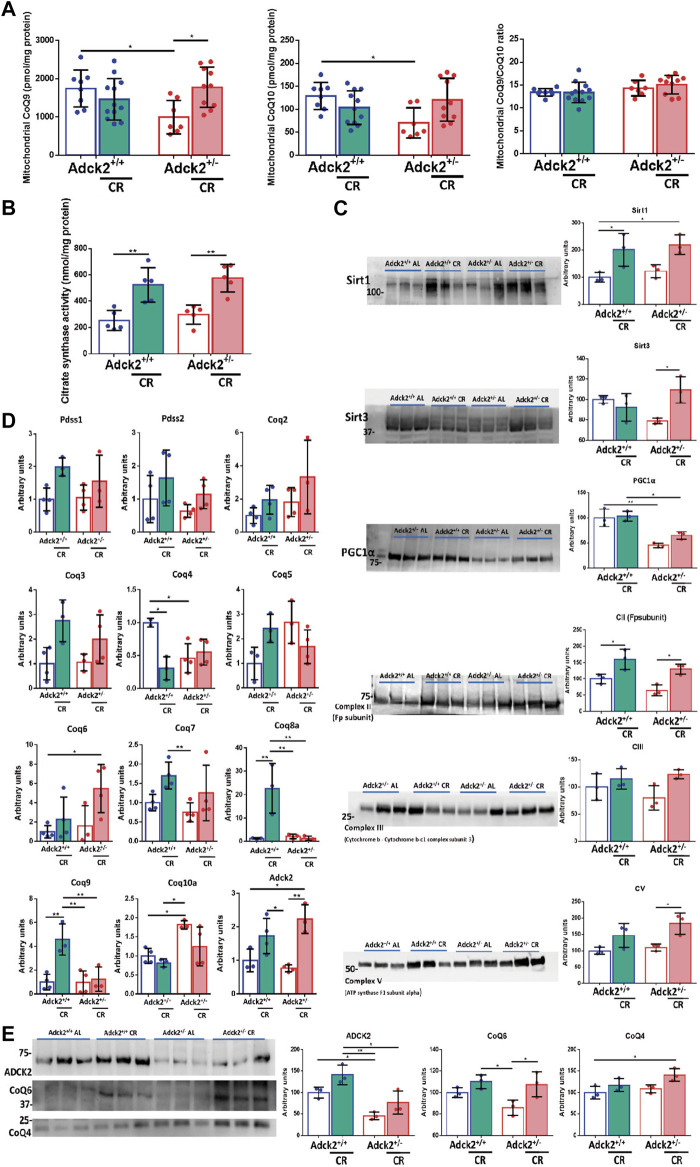
CR modulation over CoQ levels and mitochondrial biogenesis. **(A)**. CoQ_9_ and CoQ_10_ levels on mitochondria isolated from skeletal muscle. Ratio CoQ_9_/CoQ_10_. Data represents the mean ± SD (Adck2^+/+^ N = 8, Adck2^+/−^ N = 7, Adck2^+/+^ CR N = 12, Adck2^+/−^ CR N = 10) **(B)**. Citrate synthase activity on skeletal muscle. Data represents the mean ± SD N = 5 **(C)**. Protein levels of mitochondrial metabolic markers from skeletal muscle homogenate. Data represents the mean ± SD N = 3 **(D)**. Expression levels of CoQ genes in skeletal muscle homogenate. CR, calorie restriction. Data represent the mean ± SD N = 3-4. **(E)**. Adck2 and Coq proteins levels in mitochondrial isolated from skeletal muscle. CR, calorie restriction. Data represent the mean ± SD N = 3. One-way ANOVA test was applied. *p < 0.05; **p < 0.01; ***p < 0.001; ****p < 0.0001.

To explore the mechanism of CR in CoQ-defective skeletal muscle, we analyzed CoQ_9_ and CoQ_10_ levels in isolated mitochondria from both genotypes and feeding conditions. Skeletal muscle mitochondria of *Adck2* ^+/−^ mice fed on *ad libitum* condition showed significantly lower levels of CoQ_9_ and CoQ_10_ when compared to WT mice on *ad libitum* diet. CR induced a significant increase of both CoQ_9_ and CoQ_10_ in *Adck2* ^+/−^ mitochondria (Figure 8A), maintaining the ratio between both isoforms. The improvement of mitochondria metabolism observed in *Adck2* ^+/−^ mice under CR diet correlated with the increase of Citrate Synthase activity in skeletal muscle as previously shown in WT mice (Hepple et al., 2006), indicating higher mitochondrial mass in skeletal muscle from mice on CR (Figure 8B). Although, protein levels of mitochondrial mass markers were not different between WT and *Adck2* ^+/−^ mice on *ad libitum* diet, we found a general upregulation in both groups of mice on CR conditions for mitochondrial markers, including Complexes II, III and V subunits, and small increase of PGC1α protein levels (Figure 8C). Additionally, SIRT1 protein levels were upregulated in mice on CR, indicating an adequate adaptation to fasting. SIRT3 protein, the main mitochondrial deacetylase, was also increased in CR *Adck2* ^+/−^ mice (Figure 8C). To confirm that the CoQ increase was due to biosynthesis activation, gene expression of components of its biosynthetic pathway were analyzed by qPCR. CR modulated some of the genes in the pathway, including *Adck2* (Figure 8D). Furthermore, ADCK2 and other COQ protein levels were analyzed in mitochondrial fraction isolated from skeletal muscle, observing a 50% decrease in *Adck2* ^+/−^ versus WT mice on *ad libitum*, and an increase in *Adck2* ^+/−^ mice under CR (Figure 8E). Overall, CR benefits on *Adck2* ^+/−^ mice, such as activation of mitochondrial biogenesis and CoQ biosynthesis in skeletal muscle, would also explain the remodeling in bioenergetics and metabolism.

## Discussion

*ADCK2* haploinsufficiency causes mitochondrial myopathy (Vázquez-Fonseca et al., 2019) with defects in the oxidation of fatty acids in skeletal muscle, and accelerated physical deterioration in humans, and in a heterozygous *Adck2* knockout mouse model. Skeletal muscle mitochondrial dysfunction is caused by significant low levels of CoQ, decreasing oxidative phosphorylation, and also affecting important metabolic pathways, such as the pyrimidine nucleotide biosynthesis, which depends on specific CoQ reductive reactions and conditions metabolic homeostasis (Banerjee et al., 2021). *Adck2* ^+/−^ mice show a metabolic adaptation that includes insulin resistance, increased glucose catabolism, and defective mitochondrial fatty acids β-oxidation, which could impact upon liver and skeletal muscle function and plasma metabolites concentrations, fitting with the reduced physical performance and the increased weight gain described in this work. Insulin resistance has been associated with mitochondrial dysfunction (Fisher-Wellman and Neufer, 2012; Montgomery and Turner, 2015), and particularly in CoQ deficiency conditions, in which human and mouse adipose tissue show decreased CoQ biosynthesis pathway enzymes (Fazakerley et al., 2018). This fact is also evidenced in non-diabetic individuals from the Baltimore Longitudinal Study of Ageing cohort, in which a more impaired mitochondrial capacity is associated with greater insulin resistance (Fabbri et al., 2017).

Ageing modulates phenotypical, physiological, and biochemical aspects of all organisms leading to progressive loss of function, incapacity, higher disease prevalence, and ultimately death. Several markers have been linked to age-dependent frailty and decline, including physiological, functional and metabolic signs (Petr et al., 2021). The phenotype of *Adck2* mice on *ad libitum* conditions, described here, resembled the phenotype of old mice including deregulated glucose and insulin homeostasis, altered metabolic markers, decreased physical capacity, shifted skeletal muscle fiber type, and reduced in mitochondrial respiration. CR has been proved to be a powerful tool to increase lifespan and healthspan, and to delay the onset of chronic diseases associated with ageing, switching the energetic supply from glucose to fatty acids and amino acids (López-Otín et al., 2013; Ham et al., 2021). Because of this similarity, we hypothesised that *Adck2* ^+/−^ mice could benefit from CR, protecting skeletal muscle integrity during ageing (Ham et al., 2021), improving glucose and insulin sensitivity (Ham et al., 2021), mitochondrial health and morphology (Li et al., 2022), and elevating mitochondrial CoQ (Kamzalov and Sohal, 2004). Especially, as CR has shown more pronounced effects in deregulated mitochondrial metabolism conditions such as those found in ageing (Li et al., 2022). Indeed, here we show that CR can recover the *Adck2* ^+/−^ mice phenotype in terms of glucose and insulin homeostasis, physical capacity, fatty acids β-oxidation, oxidative profile in myofibers and mitochondrial respiration. We propose that changes on mitochondrial ultrastructure (Gutiérrez-Casado et al., 2019) and dynamics (Liesa and Shirihai, 2013) modulated by CR could promote a bioenergetic adaptation and a most efficient use of substrates resulting in an improvement of mitochondrial respiration and ETC function particularly in *Adck2* ^+/−^skeletal muscle.

Satellite cell function and metabolism depend on their mitochondrial activity (Forni et al., 2016; Khacho and Slack, 2017). Thus, we developed an *in vitro* approach to focus on the effect of CR over satellite cell proliferation and differentiation. Do determine if sera composition could influence the differentiation process, these cells were cultivated with serum from either mutant or WT mice fed on *ad libitum* or CR conditions. Serum composition has been shown to be crucial during differentiation of adult muscle stem cells, as myotubes growth is influenced differently by sera from young and old humans (Allen et al., 2021). Satellite cells cultivated with serum from *Adck2* ^+/−^ mice on *ad libitum* differentiated 2-3 times slower, showed lower branching capacity, MHC area, myotube length, and differentiation index, when compared to satellite cells cultivated with serum from WT on *ad libitum*. However, mutant satellite cells cultivated with serum from *Adck2* ^+/−^ mice on CR differentiated faster and showed myotube differentiation similar to that of WT satellite cells in the same conditions (Figures 6B,C), which is directly associated with the increase in oxidative pathways with better fatty acids use. During differentiation of adult muscle stem cells, a progressive metabolic shift from glycolysis to oxidative phosphorylation occurs through the activation of mitochondria and oxidative pathways (Relaix et al., 2021), although other factors could also be involved (Abreu et al., 2020). It has been previously shown that the addition of mammalian CR serum to cell cultures could result in a higher tolerance to stress response, reduced cell proliferation and lower oxidative damage due to components present in the serum (De Cabo et al., 2003; García-Matas et al., 2015; Novelle et al., 2015). Here, we propose that serum from CR mice could force activation of mitochondria, leading to increased oxidative metabolism and accelerated myotube differentiation of satellite cells.

Age-associated mitochondrial dysfunction is rescued by CR through mitochondria biogenesis and SIRT3-dependent increased respiration (López-Lluch et al., 2006; Hebert et al., 2013; Martin-Montalvo and De Cabo, 2013), which are major mechanisms to improve health span in mammals (De Cabo et al., 2014). ADCK2 haploinsufficiency in 6 month-old mice causes a pathophenotype similar to that of old mice fed *ad libitum*, which make these mutant mice suitable to benefit by CR intervention. *Adck2* ^+/−^ mice show a mitochondrial dysfunction in skeletal muscle probably driven by the defect in CoQ biosynthesis, causing defects in the electron transport chain affecting ATP production and essential metabolic pathways (Alcázar-Fabra et al., 2021; Banerjee et al., 2021). Mitochondrial dysfunction in *Adck2* ^+/−^ mice is also associated with a low oxidation of fatty acids and consequently reduced physical capacity, similarly to other mouse models defective for fatty acids transport into mitochondria (Ghosh et al., 2019; Zhao et al., 2019). It is possible that different mechanisms could be involved in the benefits generated by CR in *Adck2* ^+/−^ mice, as it has been observed in other mouse models (Mitchell et al., 2016). In this sense, CR improved all these aspects associated with mitochondrial dysfunction in skeletal muscle, which could be explained by an expression increase of remaining *Adck2* allele, increase that is associated with activation of mitochondrial biogenesis and CoQ biosynthesis pathways. Probably, CR modulates additional mechanisms as glucose homeostasis and global metabolism remodeling impact upon other organs and not only skeletal muscle.

CR induced CoQ biosynthesis in both genotypes, but we only found an increase of CoQ levels in *Adck2* ^+/−^ mitochondria. We think that wild type mitochondria were at its maximum capacity and could not store additional CoQ (Fernández-Ayala et al., 2005) which would be transferred to other cellular membranes such as the plasma membrane (De Cabo et al., 2004; López-Lluch et al., 2005; Hyun et al., 2006).

*ADCK2* haploinsufficiency can be grouped into mitochondrial diseases due to defects in nuclear-encoded genes whose encoded proteins are located inside mitochondria (Bellusci et al., 2021; Navas et al., 2021). Here, we show that most of the mitochondrial dysfunction could be alleviated by CR in a mouse model for the disease. We propose that CR should be considered an alternative for the treatment of mitochondrial diseases, and particularly for those with a mild pathology and caused by nuclear genome defects.

## Data Availability

The original contributions presented in the study are included in the article/Supplementary Material, further inquiries can be directed to the corresponding author.
