# Supplementary figures and images for: Calorie Restriction Rescues Mitochondrial Dysfunction in Adck2-Deficient Skeletal Muscle
Source: Front Physiol. 2022 Jul 14;13:898792. doi: 10.3389/fphys.2022.898792 (PMC9351392; doi:10.3389/fphys.2022.898792)

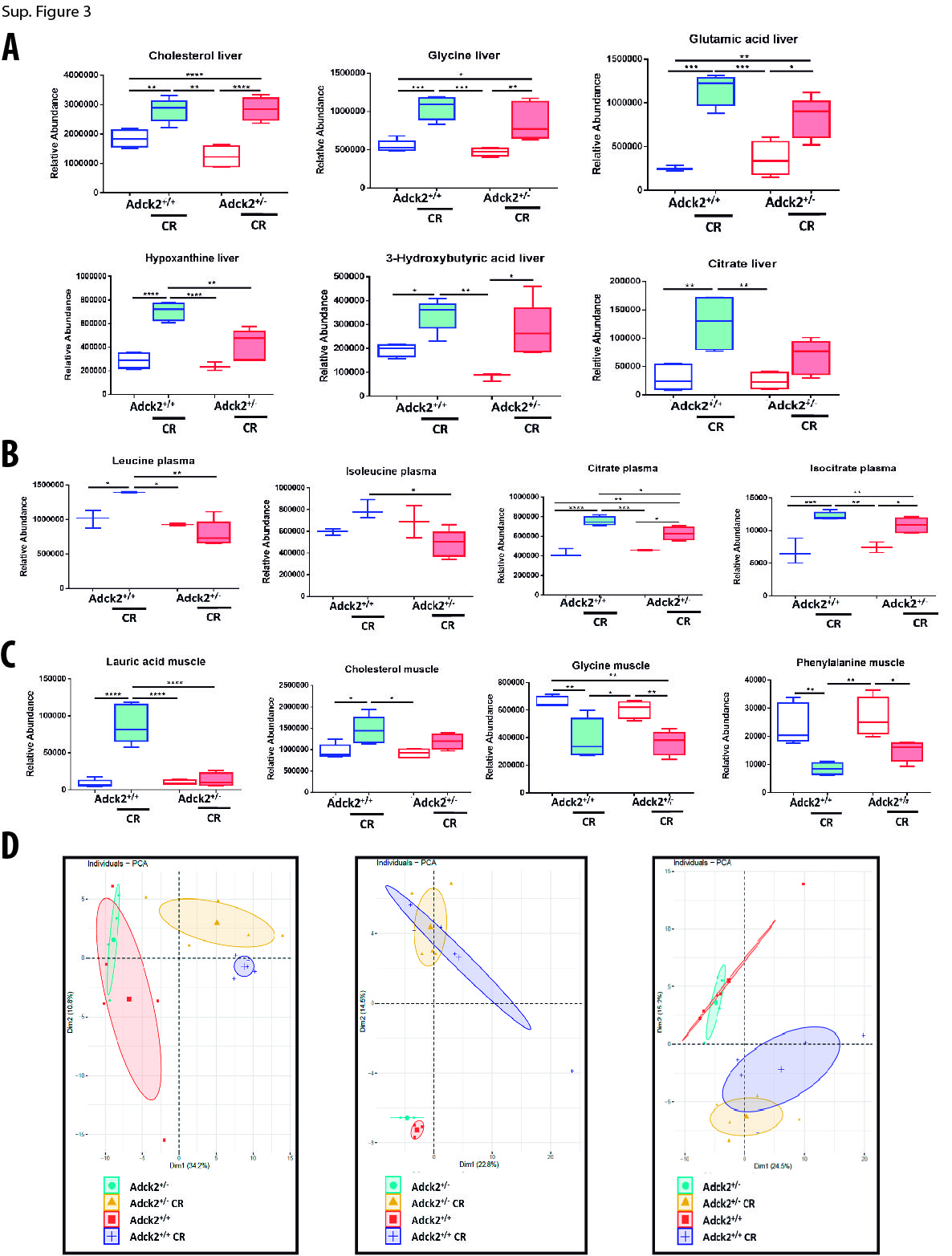

Supplement: Supplementary file 1 [file Image3.jpeg]

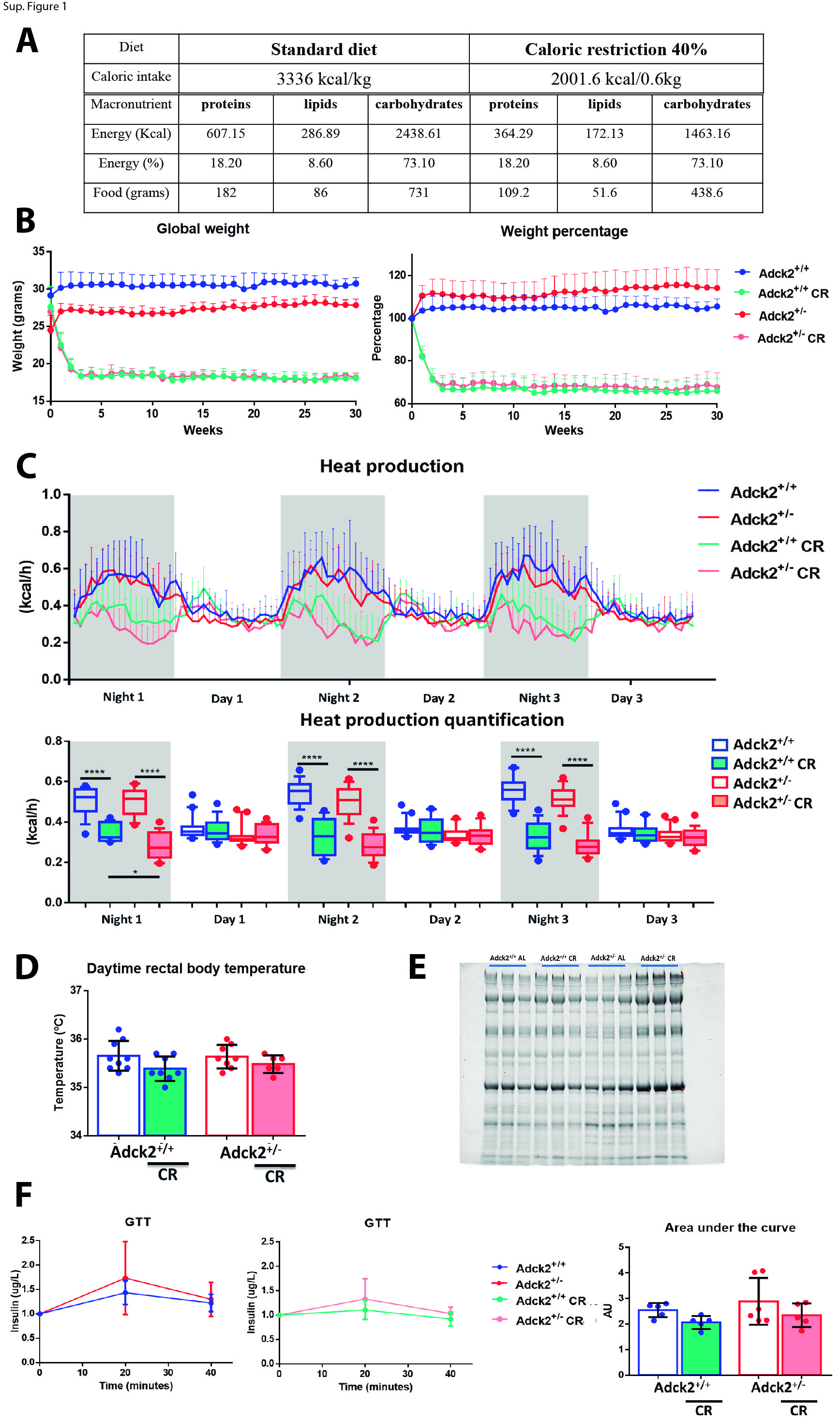

Supplement: Supplementary file 2 [file Image1.jpeg]

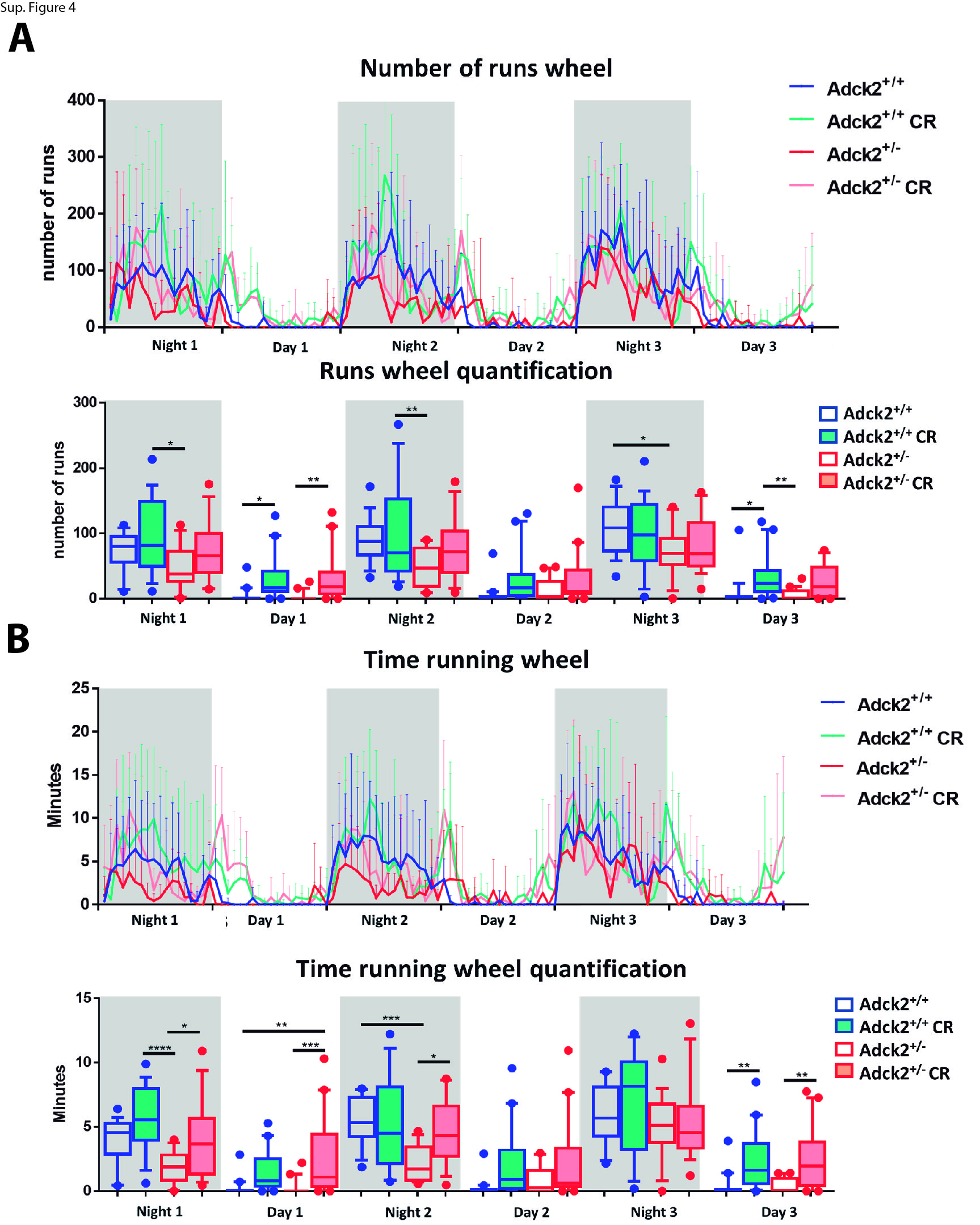

Supplement: Supplementary file 3 [file Image4.jpeg]

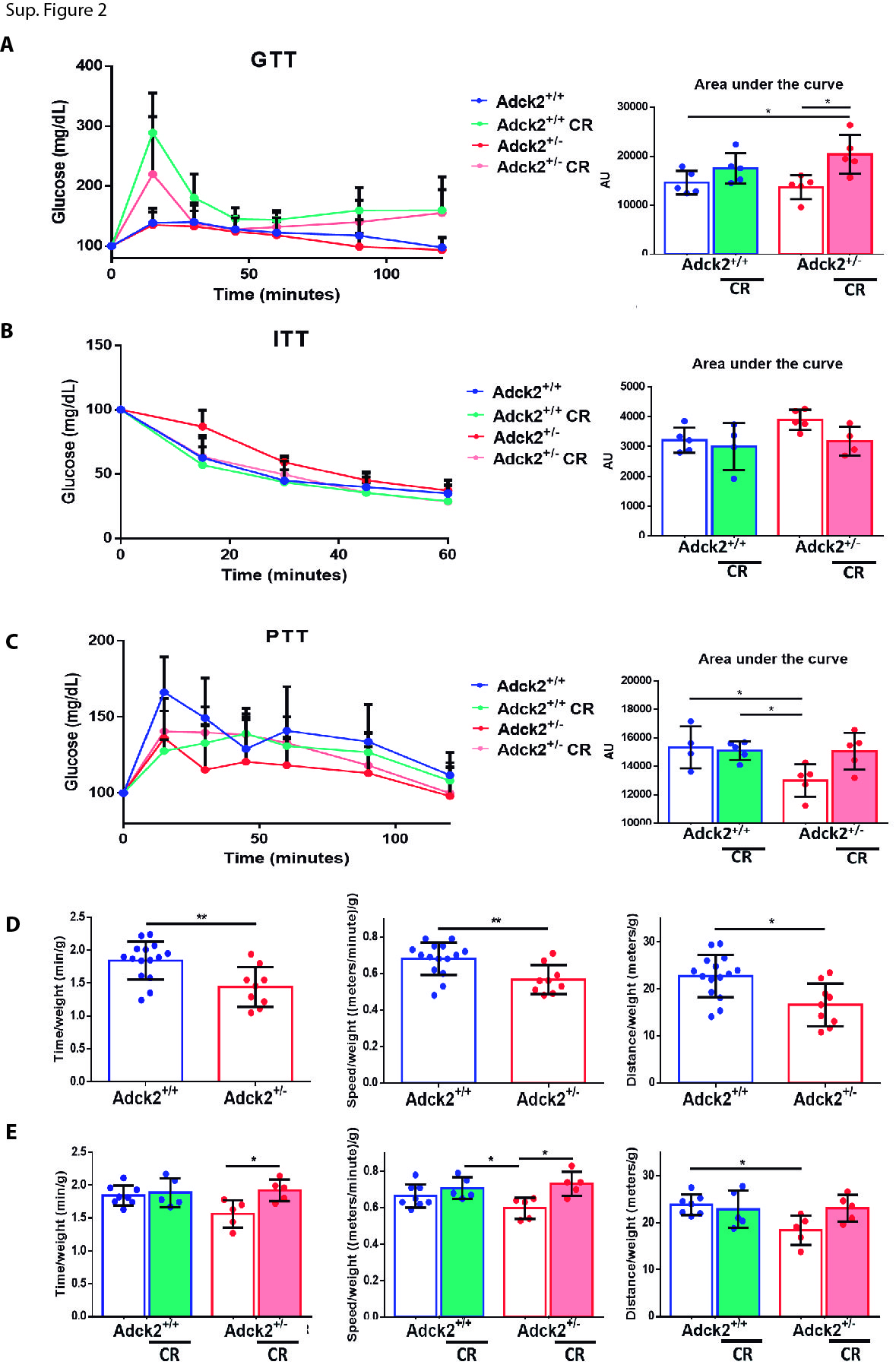

Supplement: Supplementary file 4 [file Image2.jpeg]
