# Supplementary material for: Calorie Restriction Rescues Mitochondrial Dysfunction in Adck2-Deficient Skeletal Muscle
Source: Front Physiol. 2022 Jul 14;13:898792. doi: 10.3389/fphys.2022.898792 (PMC9351392; doi:10.3389/fphys.2022.898792)
Supplement: Supplementary file 5 [file DataSheet1.docx]

**Calorie restriction rescues mitochondrial dysfunction in Adck2-deficient skeletal muscle**

Juan Diego Hernández-Camacho^1,2^, Daniel J. M. Fernández-Ayala^1,2^, Cristina Vicente-García^1^, Ignacio Navas-Enamorado^1,3^, Guillermo López-Lluch^1,2^, Clara Oliva^2,4^, Rafael Artuch^2,4^, Judith Garcia-Villoria^2,5^, Antonia Ribes^2,5^, Rafael de Cabo^6^, Jaime J. Carvajal^1^, Plácido Navas^1,2*^

^1^Centro Andaluz de Biología del Desarrollo, Universidad Pablo de Olavide-CSIC-JA, Sevilla, Spain

^2^CIBERER, Instituto de Salud Carlos III, Madrid, Spain

^3^Atsena Therapeutics, Durham, NC, USA

^4^ Clinical Biochemistry Department, Institut de Recerca Sant Joan de Déu, Barcelona, Spain

^5^Inborn Errors of Metabolism Division, Biochemistry and Molecular Genetics Department, Hospital Clinic, Barcelona, Spain

^6^Translational Gerontology Branch, National Institute on Aging Intramural Program, National Institutes of Health, Baltimore, MD, USA

*** Correspondence:**Corresponding Author: Plácido Navas
[pnavas@upo.es](mailto:pnavas@upo.es)

**Supplementary Figure 1. Weight and body temperature modulation by caloric restriction.**

(A). Diet information including calories and macronutrients intakes. Mice on CR consumed 60% of *ad libitum* food, diet information is expressed as 60% of one kilogram of *ad libitum* diet. (B). Weight analysis of WT and *Adck2*+/- on ad libitum and on CR diets along 30 weeks. Total weight for the different groups is shown in the left panel, while percentages of weight change are shown on the right. (Adck2^+/+^ N=15, Adck2^+/-^ N=15, Adck2^+/+^ CR N=15, Adck2^+/-^ CR N=7). (C). Heat production normalized by body weight for 3 day/night periods. Heat production was quantified with the automated home cage phenotyping system (N=8). (D). Daytime rectal body temperature under caloric restriction. Body temperature was checked at 12:00 pm with Surface Temperature Sensor (CHF44.00) (Adck2^+/+^ N=9, Adck2^+/-^ N=8, Adck2^+/+^ CR N=8, Adck2^+/-^ CR N=6). (E). Represetative Stain-free image used as loading control for western blot analyses. (F). Plasma insulin after glucose intraperitoneal injection. Glucose tolerance test (GTT) and Area under the curve. (Adck2^+/+^ N=5, Adck2^+/-^ N=6, Adck2^+/+^ CR N=5, Adck2^+/-^ CR N=5). Data represents the mean +/- SD. One-way ANOVA test was applied. **P*<0.05; ***P*<0.01; ****P*<0.001; *****P*<0.0001.

**Supplementary Figure 2. Glucose and insulin homeostasis on calorie restriction conditions normalized by minus baseline.**

(A). Glucose tolerance test (GTT) and area under the curve (AUC) quantification minus baseline (N=5). (B). Insulin tolerance test (ITT) and AUC quantification minus baseline (N=5). (C). Pyruvate tolerance test (PTT) and AUC quantification minus baseline (N=5). CR, calorie restriction. Data represent the mean +/- SD. Results for the aerobic treadmill tests normalized by body weight are shown on basal conditions before starting diet period (D) (Adck2^+/+^ N=15, Adck2^+/-^ N=9), and at end of diet period (E) (Adck2^+/+^ N=8, Adck2^+/-^ N=5, Adck2^+/+^ CR N=5, Adck2^+/-^ CR N=5). CR, calorie restriction. Data represent the mean +/- SD. One-way ANOVA test was applied. *P<0.05; **P<0.01; ***P<0.001; ****P<0.0001.

**Supplementary Figure 3. Functional validation and representation of metabolomics**

(A). Metabolic profile validation in liver tissue. (Adck2^+/+^ N=4, Adck2^+/-^ N=4, Adck2^+/+^ CR N=5, Adck2^+/-^ CR N=5) (B). Metabolic profile validation in plasma. (Adck2^+/+^ N=3, Adck2^+/-^ N=3, Adck2^+/+^ CR N=4, Adck2^+/-^ CR N=4). (C). Metabolic profile validation in muscle. (Adck2^+/+^ N=4, Adck2^+/-^ N=4, Adck2^+/+^ CR N=5, Adck2^+/-^ CR N=5). (D). PCA analysis on liver, plasma and muscle. One-way ANOVA test was applied. **P*<0.05; ***P*<0.01; ****P*<0.001; *****P*<0.0001.

**Supplementary Figure 4. Additional data from voluntary activity in the running wheel**

(A). Time of wheel running activity by the different mice for a 3 day/night period. Total wheel running time was quantified with the automated home cage phenotyping system. (Adck2^+/+^ N=7, Adck2^+/-^ N=5, Adck2^+/+^ CR N=7, Adck2^+/-^ CR N=8). (B). Number of wheel running events in the different groups studied for a 3 day/night period. The number of wheel running evnts quantified with the automated home cage phenotyping system. (Adck2^+/+^ N=7, Adck2^+/-^ N=5, Adck2^+/+^ CR N=7, Adck2^+/-^ CR N=8). Data represent the mean +/- SD. One-way ANOVA test was applied. P-values <0.05 were considered significant. Significant differences are indicated by asterisks (*p<0.05 ; ** p<0.01 ; ***p<0.001; ****p<0.0001).

**Supplementary Table 1. Primary antibodies**

| **Antigen** | **Mono/polyclonal** | **Host species** | **Dilution** | **Company** | **Reference** | **Application** |
| --- | --- | --- | --- | --- | --- | --- |
| MHC | Monoclonal | Mouse | 1:300 | DSHB | MF20-c | IHC |
| Myosin heavy chain Type IIB | Monoclonal | Mouse | 1:100 | DSHB | BF-F3 | IHC |
| Myosin heavy chain Type IIA | Monoclonal | Mouse | 1:600 | DSHB | SC-71 | IHC |
| AKT | Monoclonal | Rabbit | 1:1000 | CS | C67E7 | WB |
| Fp Subunit | Monoclonal | Mouse | 1:1000 | Invitrogen | 459200 | WB |
| MT-CYB | Monoclonal | Mouse | 1:1000 | Millipore | MABS2036 | WB |
| ATP5A | Monoclonal | Mouse | 1:1000 | Abcam | ab14748 | WB |
| UQCRC2 | Monoclonal | Mouse | 1:1000 | Abcam | Ab14745 | WB |
| Sirt3 | Polyclonal | Rabbit | 1:1500 | Thermo | PA5-13222 | WB |
| PGC1α | Polyclonal | Rabbit | 1:1000 | Abcam | Ab191838 | WB |
| AMPKα | Polyclonal | Rabbit | 1:1500 | SC | sc-25792 | WB |
| ERK1 | Polyclonal | Rabbit | 1:1500 | SC | sc-93 | WB |
| Foxo-1 | Polyclonal | Rabbit | 1:1000 | SC | sc-49437 | WB |
| Foxo-4 | Polyclonal | Goat | 1:1000 | SC | sc-34899 | WB |
| Sirt1 | Monoclonal | Mouse | 1:1000 | Sigma | 04-1557 | WB |
| PPARα | Monoclonal | Rabbit | 1:1500 | Thermo | MA1-822 | WB |
| AACA2 | Monoclonal | Mouse | 1:1000 | SC | 100847 | WB |
| ADCK2 | Polyclonal | Rabbit | 1:1000 | Abcam | Ab72758 | WB |
| CoQ6 | Polyclonal | Rabbit | 1:500 | Proteintech | 12481-1-AP | WB |
| CoQ4 | Polyclonal | Rabbit | 1:1000 | Proteintech | 16654-1-AP | WB |

Developmental Studies Hybridoma Bank (DSHB), Cell Signaling (CS), Santa Cruz (SC), Western blot (WB), Immunohistochemistry (IHC).

**Supplementary Table 2. Secondary antibodies**

| **Antigen** | **Host species** | **Dilution** | **Company** | **Reference** | **Function** |
| --- | --- | --- | --- | --- | --- |
| Anti-Goat | Rabbit | 1:5000 | Sigma | 401504 | HRP |
| Anti-Rabbit | Goat | 1:5000 | CS | 7074 | HRP |
| Anti-Mouse | Goat | 1:5000 | JL | 115-035-006 | HRP |
| Anti-Mouse IgG (H+L) | Goat | 1:1000 | Thermo | A-11001 | Alexa 488 |
| Anti-Mouse IgG1 | Goat | 1:500 | Thermo | A-21121 | Alexa 488 |
| Anti-Mouse IgM | Goat | 1:500 | Thermo | A-21426 | Alexa 555 |

Cell Signaling (CS), Jackson Laboratory (JL).

**Supplementary Table 3. Primers for qPCR.**

| **Target**  **genes** | **Forward (5’-3’)** | **Reverse (5’-3’)** |
| --- | --- | --- |
| **HPRT** | CAGTCAACGGGGGACATAAA | AGAGGTCCTTTTCACCAGCAA |
| **Pdss1** | CATCAAAGGACACCAGCAATGT | GCACCACAATAATCGGTCTAAAGG |
| **Pdss2** | ATGCTGACCTCCAGCCTTTT | GTCACACCTTTGCCAGCTTT |
| **Coq2** | GCCCACCAGGACAAGAAAGAC | AGCCACAGCAGCGTAGTAGG |
| **Coq3** | GTGAGCCACCTGGAAATGTT | CCCACGTATGAGTGCCTTTT |
| **Coq4** | GGGGAGACCACAGGA TGC | GTCGAGGGTAGACAGCGAGAT |
| **Coq5** | GGGCAGTTCTTCAGCGTC T | GGATTCCTTGGGAGGTTC |
| **Coq6** | CGACGTGGTGGTGTCAGC | AGTTTCTCCAGGGCTTTCTTT |
| **Coq7** | TGATGGAAGAGGACCCTGAGAAG | GCCTGTATCGTGGTGTTCAAGC |
| **Coq8** | AGCAAGCCACACAAGCAGATG | CCAGACCTACAGCCAGACCTC |
| **Coq9** | CCCGAGTTTTCCCGTCC | TGGGCTCCTTCAGCAATG |
| **Coq10** | TAAACAGAACCCTTCCACCG | CGAAATGCTGATAGTCCTCCA |
| **Adck2** | TGGTGGGTCAGAAGTGGGTGTGTC | GGGTCTCTTTCGGATCTGGGGCAG |

**Supplementary Table 4. Statistical analysis of metabolites.**

| **Metabolomics on liver** | | | **Statistical analysis** | | | |
| --- | --- | --- | --- | --- | --- | --- |
| Comparison | Adck2^+/+^ vs Adck2^+/+^ CR | Adck2^+/+^ vs Adck2^+/-^ | Adck2^+/+^ vs Adck2^+/-^ CR | Adck2^+/+^ CR vs Adck2^+/-^ | Adck2^+/+^ CR vs Adck2^+/-^ CR | Adck2^+/-^ vs Adck2^+/-^ CR |
| Metabolite |  |  |  |  |  |  |
| Myristate acid | **** | ** | ns | ns | **** | ** |
| Adipic acid | ns | ns | ns | * | ns | ns |
| Cholesterol | ** | ns | **** | ** | ns | **** |
| Glycine | *** | ns | * | *** | ns | ** |
| Phenylalanine | * | ns | ns | * | ns | ns |
| Tyrosine | ** | ns | ns | ** | ns | * |
| Isoleucine | ** | ns | * | * | ns | ns |
| Leucine | **** | ns | * | **** | ** | ns |
| Threonine | * | ns | ns | * | ns | ns |
| Aspartic acid | *** | ns | * | ** | ns | * |
| Methionine | ns | ns | ns | ** | ns | ns |
| Proline | *** | ns | ns | *** | * | ns |
| Alanine | ns | ns | ns | ns | ns | * |
| Valine | ** | ns | ns | ** | * | ns |
| Glutamic acid | *** | ns | ** | *** | ns | * |
| Glutamine | ns | ns | ** | ns | * | * |
| Hypoxanthine | **** | ns | ns | **** | ** | ns |
| 3-Hydroxybutiric acid | * | ns | ns | ** | ns | * |
| Citrate | ** | ns | ns | ** | ns | ns |
| Ornithine | ** | ns | ns | * | ns | ns |
| Glucose | * | ns | ns | ** | ns | ns |
| Lactate | * | ns | ns | ** | ns | ns |
| **Metabolomics on skeletal muscle** | | | **Statistical analysis** | | | |
| Comparison | Adck2^+/+^ vs Adck2^+/+^ CR | Adck2^+/+^ vs Adck2^+/-^ | Adck2^+/+^ vs Adck2^+/-^ CR | Adck2^+/+^ CR vs Adck2^+/-^ | Adck2^+/+^ CR vs Adck2^+/-^ CR | Adck2^+/-^ vs Adck2^+/-^ CR |
| Metabolite |  |  |  |  |  |  |
| Linoleic acid | *** | ns | ns | * | * | ns |
| Cholesterol | * | ns | ns | * | ns | ns |
| Glycine | ** | ns | ** | * | ns | ** |
| Threonine | * | ns | ns | * | ns | ns |
| Leucine | ns | ns | ns | ** | ns | * |
| Isoleucine | ** | ns | * | *** | ns | *** |
| Tyrosine | ns | ns | ** | ns | ns | ns |
| Methionine | ** | ns | ** | ** | ns | ** |
| Proline | ns | ns | ns | ns | ns | ** |
| Serine | ns | ns | ns | * | ns | * |
| 3-Hydroxybutiric acid | ns | ns | ns | * | ns | ns |
| Citrate | ** | ns | *** | ns | ns | * |
| 2-hydroxy-glutarate | ns | ns | *** | ns | ** | **** |
| **Metabolomics on plasma** | | | **Statistical analysis** | | | |
| Comparison | Adck2^+/+^ vs Adck2^+/+^ CR | Adck2^+/+^ vs Adck2^+/-^ | Adck2^+/+^ vs Adck2^+/-^ CR | Adck2^+/+^ CR vs Adck2^+/-^ | Adck2^+/+^ CR vs Adck2^+/-^ CR | Adck2^+/-^ vs Adck2^+/-^ CR |
| Metabolite |  |  |  |  |  |  |
| Leucine | * | ns | ns | * | ** | ns |
| Valine | * | ns | ns | ns | ** | ns |
| Phenylalanine | ns | ns | ns | ns | ** | ns |
| Isoleucine | ns | ns | ns | ns | * | ns |
| Trytophan | * | ns | ns | ns | ns | ns |
| 2-hydroxy-glutarate | *** | ns | **** | *** | ns | **** |
| Citrate | *** | ns | ** | *** | * | * |
| Isocitrate | *** | ns | ** | ** | ns | * |

**Supplementary Table 5. Statistical analysis of branching capacity**

| **Time point 24 hours** | | | | | |
| --- | --- | --- | --- | --- | --- |
| **Branches 0** | | **Branches 1** | | **Branches 2** | |
| **Comparison** | **Significance** | **Comparison** | **Significance** | **Comparison** | **Significance** |
| Adck2^+/+^ vs Adck2^+/+^ CR | * | Adck2^+/+^ vs Adck2^+/+^ CR | * | Adck2^+/+^ vs Adck2^+/+^ CR | ns |
| Adck2^+/+^ vs Adck2^+/-^ | ns | Adck2^+/+^ vs Adck2^+/-^ | ns | Adck2^+/+^ vs Adck2^+/-^ | ns |
| Adck2^+/+^ vs Adck2^+/-^ CR | ns | Adck2^+/+^ vs Adck2^+/-^ CR | * | Adck2^+/+^ vs Adck2^+/-^ CR | ns |
| Adck2^+/+^ CR vs Adck2^+/-^ | ns | Adck2^+/+^ CR vs Adck2^+/-^ | ns | Adck2^+/+^ CR vs Adck2^+/-^ | ns |
| Adck2^+/+^ CR vs Adck2^+/-^ CR | ns | Adck2^+/+^ CR vs Adck2^+/-^ CR | ns | Adck2^+/+^ CR vs Adck2^+/-^ CR | ns |
| Adck2^+/-^ vs Adck2^+/-^ CR | ns | Adck2^+/-^ vs Adck2^+/-^ CR | ns | Adck2^+/-^ vs Adck2^+/-^ CR | ns |
| **Time point 48 hours** | | | | | |
| **Branches 0** | | **Branches 1** | | **Branches 2** | |
| **Comparison** | **Significance** | **Comparison** | **Significance** | **Comparison** | **Significance** |
| Adck2^+/+^ vs Adck2^+/+^ CR | ns | Adck2^+/+^ vs Adck2^+/+^ CR | ns | Adck2^+/+^ vs Adck2^+/+^ CR | ns |
| Adck2^+/+^ vs Adck2^+/-^ | ns | Adck2^+/+^ vs Adck2^+/-^ | ns | Adck2^+/+^ vs Adck2^+/-^ | ns |
| Adck2^+/+^ vs Adck2^+/-^ CR | ns | Adck2^+/+^ vs Adck2^+/-^ CR | ns | Adck2^+/+^ vs Adck2^+/-^ CR | ns |
| Adck2^+/+^ CR vs Adck2^+/-^ | ns | Adck2^+/+^ CR vs Adck2^+/-^ | ns | Adck2^+/+^ CR vs Adck2^+/-^ | ns |
| Adck2^+/+^ CR vs Adck2^+/-^ CR | ns | Adck2^+/+^ CR vs Adck2^+/-^ CR | ns | Adck2^+/+^ CR vs Adck2^+/-^ CR | ns |
| Adck2^+/-^ vs Adck2^+/-^ CR | * | Adck2^+/-^ vs Adck2^+/-^ CR | ns | Adck2^+/-^ vs Adck2^+/-^ CR | ns |
| **Time point 72 hours** | | | | | |
| **Branches 0** | | **Branches 1** | | **Branches 2** | |
| **Comparison** | **Significance** | **Comparison** | **Significance** | **Comparison** | **Significance** |
| Adck2^+/+^ vs Adck2^+/+^ CR | * | Adck2^+/+^ vs Adck2^+/+^ CR | ns | Adck2^+/+^ vs Adck2^+/+^ CR | * |
| Adck2^+/+^ vs Adck2^+/-^ | * | Adck2^+/+^ vs Adck2^+/-^ | ns | Adck2^+/+^ vs Adck2^+/-^ | ns |
| Adck2^+/+^ vs Adck2^+/-^ CR | *** | Adck2^+/+^ vs Adck2^+/-^ CR | ns | Adck2^+/+^ vs Adck2^+/-^ CR | ** |
| Adck2^+/+^ CR vs Adck2^+/-^ | **** | Adck2^+/+^ CR vs Adck2^+/-^ | ** | Adck2^+/+^ CR vs Adck2^+/-^ | ** |
| Adck2^+/+^ CR vs Adck2^+/-^ CR | ns | Adck2^+/+^ CR vs Adck2^+/-^ CR | ns | Adck2^+/+^ CR vs Adck2^+/-^ CR | ns |
| Adck2^+/-^ vs Adck2^+/-^ CR | **** | Adck2^+/-^ vs Adck2^+/-^ CR | ** | Adck2^+/-^ vs Adck2^+/-^ CR | **** |

**Supplementary Table 6. Differentiation analysis in myotubes on caloric restriction conditions.**

| **Parameter: MHC area** | | |
| --- | --- | --- |
| Group | Slope of the differentiation line | R squared on the model |
| Adck2^+/+^ *ad libitum* serum | 0.1444x | R² = 0.7640 |
| Adck2^+/+^ CR serum | 0.1600x | R² = 0.5363 |
| Adck2^+/-^ *ad libitum* serum | 0.0960x | R² = 0.5793 |
| Adck2^+/-^ CR serum | 0.1814x | R² = 0.5098 |
| **Parameter: Differentiation Index** | | |
| Group | Slope of the differentiation line | R squared on the model |
| Adck2^+/+^ *ad libitum* serum | 0.1460x | R² = 0.6411 |
| Adck2^+/+^ CR serum | 0.1801x | R² = 0.5497 |
| Adck2^+/-^ *ad libitum* serum | 0.0564x | R² = 0.1649 |
| Adck2^+/-^ CR serum | 0.1767x | R² = 0.4671 |
| **Parameter: Myotubes length** | | |
| Group | Slope of the differentiation line | R squared on the model |
| Adck2^+/+^ *ad libitum* serum | 2.2595x | R² = 0.8441 |
| Adck2^+/+^ CR serum | 3.0836x | R² = 0.8406 |
| Adck2^+/-^ *ad libitum* serum | 1.8805x | R² = 0.8600 |
| Adck2^+/-^ CR serum | 3.4944x | R² = 0.8647 |
| **Parameter: Branching capacity** | | |
| Group | Slope of the differentiation line | R squared on the model |
| Adck2^+/+^ *ad libitum* serum | 0.4157x | R² = 0.8473 |
| Adck2^+/+^ CR serum | 0.4289x | R² = 0.7696 |
| Adck2^+/-^ *ad libitum* serum | 0.2099x | R² = 0.6369 |
| Adck2^+/-^ CR serum | 0.5057x | R² = 0.8452 |
